# Phytochemical and biological activities of some Iranian medicinal plants

**DOI:** 10.1080/13880209.2022.2046112

**Published:** 2022-03-29

**Authors:** Salome Dini, Qihe Chen, Faezeh Fatemi, Younes Asri

**Affiliations:** a Department of Food Science and Nutrition, Zhejiang University, Hangzhou, China; b Young Researchers and Elite Club, Karaj Branch, Islamic Azad University, Karaj, Iran; c Nuclear Fuel Cycle Research School, Nuclear Science and Technology Research Institute, Tehran, Iran; d Research Institute of Forests and Rangelands, Agricultural Research, Education and Extension Organization (AREEO), Tehran, Iran

**Keywords:** Iranian plant species, traditional uses, volatile oils, Apiaceae, Asteraceae, Lamiaceae, Rosaceae, chemical compounds, natural antibacterial agents

## Abstract

**Context:**

Due to adverse effects of synthetic compounds, there is a growing interest in utilization of plant-derived natural products in the pharmaceutical and food industries. Iranian endemic medicinal plants widely used in traditional practice have attracted much attention as antibacterial and antioxidant agents.

**Objective:**

This review attempts to compile the accessible scientific research pertained to phytochemical compounds, antibacterial and antioxidant effects of essential oils obtained from some of the most widely used and distributed medicinal plants in Iran.

**Methods:**

This review has been compiled using references via reliable databases (Google Scholar, SID and Science Direct) from 2010 to 2020. This literature review was limited to references published in English and Persian languages.

**Results:**

Based on studies heretofore carried out, essential oils isolated from mentioned medicinal plants exhibited strong antioxidant activity which is attributed to their main phytochemical compounds; thymol, carvacrol, *p*-cymene and γ-terpinene. In addition, the antibacterial activities of essential oils of most plant species from Apiaceae and Asteraceae families were more susceptible against Gram-positive bacteria; *Staphylococcus aureus* and *Bacillus cereus* than Gram-negative bacteria; however, essential oils of other studied plant species manifested similar behaviours against both Gram-positive and -negative bacteria.

**Conclusions:**

As there is rich ethnobotanical knowledge behind Iranian endemic medicinal plants, further scientific research is required to prove their safety and efficacy. This review revealed that there are numerous valuable medicinal plants adoptable in food and pharmaceutical industries in the near future.

## Introduction

Traditional or complementary medicine is an important and often underestimated segment of health services. Nevertheless, this kind of medicine is to date acknowledged, as preferred primary health care system in many rural communities, particularly due to its affordability and effectiveness. Moreover, not only does it have a long history of use in health maintenance and disease prevention, but also an effective remedy for chronic diseases. It is estimated that over 75% of globe population rely on traditional medicines to treat various diseases, viz., diarrhoea, malaria, stomach-ache, cough, bilharzia and dysentery (Ghasemi Pirbalouti et al. 2010a; Rakotoarivelo et al. 2011; Mahomoodally and Chintamunnee 2012; Pan et al. 2014; Arumugam et al. 2016). It is even estimated that almost 25% of modern and 60% of antitumor drugs be derived from natural products (Newman and Cragg 2012; Veeresham 2012; Iqbal et al. 2017). The popularity of plant usage in Traditional Iranian Medicine (Persian Medicine) goes back to Babylonian-Assyrian civilization era (Amirmohammadi et al. 2014; Buso et al. 2020).

In spite of modern medicine development, medicinal plants still play an important role in Iran as curatives for various health problems (Akbarzadeh et al. 2015; Buso et al. 2020). Iran with different climatic and geographical zones is a habitat to at least 2300 species having aromatic and medicinal properties, wherein 7.9% are endemic (Owfi and Safaian 2017; Sheibani et al. 2018; Karim et al. 2020). Almost all parts of these plants (e.g., leaves, flowers, stems, seeds, fruits and roots) (Najafi et al. 2010; Hariri et al. 2018) produce essential oils as secondary metabolites for varied purposes; plant defence against pests or pathogens, pollinator attraction and seed dispersers (Dhifi et al. 2016). Currently, essential oils are in high demand in pharmaceutical, cosmetic, sanitary and food industries, as well as agriculture due to flavour, fragrances and versatile biological properties like antimicrobial, anticancer and antioxidant (Kumar et al. 2018). These characteristics are accredited to the presence of a complex mixture of aromatic compounds; terpenes, phenolic and phenylpropanoid compounds (Bakkali et al. 2008; Dhifi et al. 2016).

Over the past few years, antimicrobial resistance has become one of the most serious international public health concerns that threatens the effective prevention and treatment of infections resulting from a wide range of pathogens, viz., bacteria, fungi and viruses (Avaei et al. 2015; Prestinaci et al. 2015). Additionally, reducing the popularity of synthetic compounds among consumers has caused a higher discovery of natural antimicrobial agents. Generally, the mechanism of essential oil inhibiting pathogens growth is associated with essential oil type and microbial strains tested (Pauli and Kubeczka 2010). Gram-positive bacteria are generally more susceptible to essential oils than Gram-negative bacteria (Borges et al. 2013, 2014), because they are surrounded by an outer membrane which has a more complex and assisting penetration of hydrophobic compounds through it. In other words, minute antimicrobial agents can easily access the cell membrane of Gram-positive bacterial strains (Zinoviadou et al. 2009; Hyldgaard et al. 2012). Furthermore, Gram-positive bacteria may ease the infiltration of essential oils of hydrophobic compounds due to lipophilic ends of lipoteichoic acid present in the cell membrane (Cox et al. 2000). The antimicrobial activity of essential oils is commonly evaluated via minimum bactericidal concentration (MBC) or minimum inhibitory concentration (MIC) (Balouiri et al. 2016), and agar well diffusion (Rao et al. 2019).

In addition, we have noted a rise in research on the substitution of synthetic or artificial antioxidants with natural compounds since butylated hydroxyanisole and butylated hydroxytoluene (BHT) were suspected to induce carcinogenesis and liver toxicity (Caleja et al. 2017). Sequentially, adoption of essential oils as natural antioxidants in food and pharmaceutical industries has increased (Chrysargyris et al. 2020). These compounds shield human body against oxidative stress disrupt by maintaining the balance between free radicals and antioxidant defence system (Alfadda and Sallam 2012). Free radicals through oxidative stress are involved in several health disorders; cardiovascular, inflammatory, age-related diseases, cataracts and cancer (Poljsak et al. 2013). These free radicals are collectively termed reactive oxygen species (ROS) and reactive nitrogen species including highly reactive species; hydroxyl (OH·) and nitric oxide (NO·) radicals (Li et al. 2016). They are produced when our cells create energy from food and oxygen or are exposed to microbial infections, extensive exercise or pollutants/toxins, i.e., cigarette smoke, alcohol, ionizing/UV radiations, pesticides and ozone (Gilca et al. 2007). Excessive ROS generated under abiotic stress causes significant damage to biomolecules; lipids, proteins and deoxyribonucleic acid (Sharma et al. 2012) leading to different chronic diseases (Ighodaro and Akinloye 2018). The most common methods to investigate antioxidant efficacy of essential oils are ferric reducing antioxidant power (FRAP) (Benzie and Strain 1996), 2,2-diphenyl-1-picrylhydrazyl radical (DPPH) (Bondet et al. 1997), β-carotene-linoleic acid (linoleate) (Miller 1971) and 2,2-azino-bis-3-ethylbenzothiazoline-6-sulfonic acid (ABTS) assays (Bertrand et al. 2005).

Several authors have reviewed the beneficial uses of essential oils (Amorati et al. 2013; Chouhan et al. 2017). Swamy et al. (2016) reviewed selected essential oils from different countries that have great antimicrobial properties. However, few comprehensive reviews have been published on phytochemical compounds and pharmaceutical effects of Iranian endemic plants. Therefore, this review aimed to focus on 23 medicinal plants native to Iran, which are widely distributed and used in Iranian traditional medicine and among locals. The entire data were classified in three tables; traditional uses (Table 1), antioxidant effects (Table 2) and antimicrobial potential (Table 3) of essential oils from the selected medicinal plants. All the available information was compiled via reliable electronic databases; ‘Google Scholar’, ‘Science Direct’ and ‘SID’ from 2010 to 2020 to provide a foundational knowledge guide for its subsequent research and utilization.

**Table 1.** Persian names, traditional therapeutic uses and distribution of some important Iranian medicinal plants.

Scientific name	Family	Local name	Used parts of the plant	Local names/regions	Traditional uses	Ref.
*Bunium persicum* Boiss.	Apiaceae	Gharah zireh, Black zira, Zireh Kermani	Aerial parts Fruits	Qazvin, Semnan, Kerman, Khorasan, Isfahan	Indigestion, flavouring, carminative, diuretic, digestive disorders, asthma, anticonvulsant, antihelmintic, stomach disorders, liver and kidney tonic, appetizer, carminatives, antidiarrheal, colic pain, dysmenorrhoea, urinary tract disorders, emmenagogue, anticonvulsant, antihelmintic, anti-flatulent, analgesic, curing geophagy, hiccup, asphyxia, dyspnoea, spleen oedema, nasal bleeding, eye diseases, toothache	Amiri et al. (2012); Pezhmanmehr et al. (2009)
*Carum carvi* L.	Apiaceae	Zeerah Siyah	Fruits	Kerman, Yazd	Obesity, facilitate digestion, sour stomach, blood pressure, diarrhoea, laxative, carminative, appetite stimulant, lactation enhancer, menstrual pain reliever	Amiri and Joharchi (2016); Keshavarz et al. (2012); Haidari et al. (2011)
*Chaerophyllum macropodum* Boiss.	Apiaceae	Garkava, Chelghaba	Aerial parts	Kohghiluyeh va Boyer Ahmad	Cold, stomachic, culinary uses	Jahantab et al. (2018); Moazzami Farida et al. (2018)
*Cuminum cyminum* L.	Apiaceae	Zireh-Sabz	Fruits	Kerman	Carminative, obesity, digestive disorders, favouring, epilepsy, diabetes, pains	Amiri and Joharchi (2016); Gachkar et al. (2007); Johri (2011); Srinivasan (2018)
*Ferulago angulata* (Schlecht.) Boiss.	Apiaceae	Chavil-eshevidi, Chavil	Aerial parts Seeds	Khuzestan, Kermanshah, Kurdestan, Kohgiluyeh va Boyer Ahmad, Ilam, Lorestan, Fars, Markazi, Hamadan, Khorasan	Anti-septic, spice and air fresher, sedative, digestive, intestinal, worms, tonic, food-digestive, antiparasitic	Ghasemi et al. (2013); Bagherifar et al. (2019); Hazrati et al. (2019)
*Heracleum persicum* Desf.	Apiaceae	Golpar	Fruit Flowers	Mazandaran, Tehran, Qazvin	Hiccup, appetizer, flavouring, carminative, anthelmintic, stomach tonic, tremor, migraine, headache	Amiri and Joharchi (2016)
*Prangos ferulacea* (L.) Lindl.	Apiaceae	Djashir, Jâshir	Roots Leaves	Khuzestan	Wound healing, laxative, antihypertensive, carminative, digestive disorders, flavouring, animal fodder	Yousefi et al. (2017)
*Achillea millefolium* L.	Asteraceae	Ghurtgharan, Boomadaran	Inflorescence Aerial parts	Golestan, Ilam	Anthelmintic, anti-infections, wounds, antihemorrhage, stomach ache and menstrual, anti-inflammation, antidiabetic, pains, dysmenorrhoeal, diarrhoea, stomach cramps, flatulence, gastritis and gastrointestinal disturbances	Mirdeilami et al. (2011); Bahmani et al. (2014); Mazandarani et al. (2013)
*Seriphidium kermanense* (D. Podl.) Y. R. Ling [syn: *Artemisia kermanensis* Podl.]	Asteraceae	Dermaneh	Aerial parts	Kerman	Decrease blood pressure, appetizer, spice, skin disease	Dolatkhahi et al. (2014); Mozafarian (1996)
*Dracocephalum kotschyi* Boiss.	Lamiaceae	Badrandjboie-Dennaie Zarrin-Giah	Aerial parts	Mazandaran, Tehran, Isfahan, North Khorasan, Lorestan, Azerbaijan, Fars	Stomach, liver disorders, headache, congestion painkillers, kidney complications, toothaches, colds, antispasmodic	Heydari et al. (2019)
*Hymenocrater calycinus* (Boiss.) Benth.	Lamiaceae	Gol-e-Arvaneh	Aerial parts	Mazandaran, Golestan, Mazandaran, Semnan, Khorasan, Tehran, Alborz, Isfahan	Analgesic drug, skin antiallergenic, burns	Asri et al. (2017)
*Hymenocrater longiflorus* Benth.	Lamiaceae	Gole Arvaneh-Avarmani SoorSanduo	Aerial parts	Kurdestan, Kermanshah	Anti-inflammatory, sedative, anti-skin allergic reaction	Taherpour et al. (2011)
*Mentha piperita* L.	Lamiaceae	Nana felfeli	Aerial parts	Kermanshah, Khorasan, Fars	Carminative, anti-inflammatory, cold, disinfectant antispasmodic, antiemetic, diaphoretic, analgesic, stimulant, emmenagogue, anti-nausea, bronchitis, flatulence, anorexia, ulcerative colitis, liver complaints	Taherpour et al. (2017); Mikaili et al. (2012)
*Salvia mirzayanii* Rech.f. & Esfand.	Lamiaceae	Salvii, Moor, Talkh	Aerial parts	Keman, Hormozgan	Alzheimer, stomach ache, infections, spasms, gastrointestinal disorders, astringent, carminative, antiseptic, anti-diabetic, anti-inflammatory, spasmolytic, carminative, antiseptic, astringent, stomach pain	Sadat-Hosseini et al. (2017); Asadollahi et al. (2019)
*Satureja* spp.	Lamiaceae	Marzeh	Aerial parts	Khorasan, Lorestan, Ilam, Khuzetan, Fars	Indigestion, anthelmintic, appetizer, antacid, antidiarrheal, stomach-ache	Buso et al. (2020); Razzaghi-Abyaneh et al. (2013); Jamzad (1996)
*Thymus daenensis* Celak.	Lamiaceae	Avishan-e-denaee	Aerial parts	Isfahan, Fars, Chaharmahal and Bakhtiari, Lorestan, Kohgiluyeh and Boyer-Ahmad, Tehran, Isfahan, Markazi	Flavouring agents, tonic, carminative, digestive, antispasmodic, anti-inflammatory, expectorant, colds, cough, anti-bacterial, carminative	Rahimmalek et al. (2009); Emami Bistgani and Sefidkon (2019)
*Thymus kotschyanus* Boiss. & Hohen	Lamiaceae	Avishan	Aerial parts	Fars, Ardabil, The East Azerbaijan, Tehran, Yazd, Mazandaran, Hamedan	Gastrodynia, joints pain common cold, flatulence, bone pain, redness eyes, blood depurative, stomach tonic, antiseptic coughing, appetizer, kidney stones, diuretic, analgesic, high blood pressure uterine pains, headache, vomiting, heartburn, asthma, catarrh, inflammation, irritation of urinary organs, expectorant, emmenogogue, spasm, vermifuge, sedative, diaphoretic	Naghibi et al. (2005)
*Zataria multiflora* Boiss.	Lamiaceae	Avishan-e-Shirazi	Leaves Flowers	Kerman, Fars, Isfahan, Yazd, Hormozgan, Khorasan	Constipation, stomach pain menstrual cramps, cold, diarrhoea, stomach-ache, carminative, chest pain, headache, toothache, wound healing, fatigue, antipyretic, bone pain, earache, measles, reducing blood lipid and glucose	Nasab and Khosravi (2014); Safa et al. (2013)
*Zhumeria majdae* Rech.f. & Wendelbo	Lamiaceae	Moorkhash, Marvkhash, Moorkhosh	Leaves	Hormozgan	Stomach-ache antiseptic, carminative painful menstruation	Sajed et al. (2013); Rechinger (1982); Rechinger and Wendelbo (1967); Safa et al. (2013)
*Ziziphora clinopodioides* Lam.	Lamiaceae	Kakuti-e kuhi	Aerial part Roots	Yazd, Isfahan, Khorasan	Digestive system, toothache, spice	Amiri et al. (2019)
*Rosa damascena* P. Mill.	Rosaceae	Gole mohammadi	Flowers	Kashanm, Kerman	Burns and wounds healing, sedative, stomach and reflux, laxative, anti-haemorrhoid, calmative	Amiri and Joharchi (2013)

**Table 2.** Antioxidant activity of Iranian essential oils; part used, major chemical compounds and activity.

Scientific name	Part used	Major compounds	Activity	Ref.
*Bunium persicum* Boiss.	Seeds	Cuminaldehyde, carvacrol, anisole	Lower than BHT	Aminzare et al. (2017)
	Fruits	*p*-Cymene, cuminaldehyde, γ-terpinene	Much lower than vitamin C	Nickavar et al. (2014)
*Carum carvi* L.	Seeds	Cumin aldehyde γ-terpinen-7-al cumin aldehyde	Higher than BHT	Fatemi et al. (2011)
*Chaerophyllum macropodum* Boiss.	Aerial parts	*Trans*-ocimene, *cis*-ocimene, γ-terpinene	Much lower than BHT	Haghi et al. (2010)
	Aerial parts	Myrcene, (*e*)-β-ocimene, terpinolene, (*z*)-β-ocimene	Much lower than BHT	Khajehie et al. (2017)
*Cuminum cyminum* L.	Seeds	β-Pinene, γ-terpinene, cumin aldehyde, *p*-cymene	Higher than vitamin C	Fatemi et al. (2013)
	Seeds	Thymol, γ-terpinene, β-pinene	Higher than Trolox	Ladan Moghadam (2016)
*Ferulago angulata* (Schlecht.) Boiss.	Aerial parts	α-Pinene, *z*-β-ocimene	Much lower than quercetin	Shahbazi et al. (2016)
	Aerial parts	α-Pinene, *cis*-β-ocimene	Lower than BHT	Ghasemi Pirbalouti et al. (2016)
*Heracleum persicum* Desf.	Aerial parts	(*e*)-Anethole, octyl-2-methyl butanoate, octyl-2-methyl butanoate, hexyl butanoate	Much lower than quercetin	Firuzi et al. (2010)
*Prangos ferulacea* (L.) Lindl	Leaves	p-Cymene, limonene, (*e*)-β-ocimene, terpinolene, 2,3,6-trimethylbenzaldehyde	Much lower than BHT	Seidi Damyeh and Niakousari (2016)
	Flowers leaves	α-Pinene, camphene, bornylacetate	Lower than BHT	Bazdar et al. (2018)
*Achillea millefolium* L.	Aerial parts	Limonene, α-pinene, borneol, thymol, carvacrol	Higher than Trolox	Kazemi (2015)
	Aerial parts	Thymol, carvacrol	Higher than Trolox	Sahari Moghadam et al. (2017)
*Seriphidium kermanense* (D. Podl.) Y. R. Ling [syn: *Artemisia kermanensis* Podl.]	Aerial parts	Isoborneol, camphor, *cis*-thujone	Lower than BHT	Kazemi et al. (2011)
*Dracocephalum kotschyi* Boiss.	Aerial parts	α-Pinene, geranial, geranyl acetate	Higher than vitamin C	Ashrafi et al. (2017)
*Hymenocrater longiflorus* Benth.	Stems	α-Pinene, 1,8-cineole linalool *p*-menth-1-en-8-ol, β-bourbonene, *trans*-caryophyllene	Equivalent to vitamin C	Ahmadi et al. (2010)
*Mentha piperita* L.	Aerial parts	Menthol, menthofuran, 1s-neomenthyl acetate	A bit lower than BHT	Yazdani et al. (2019)
	Aerial parts	Menthol, menthone	Much lower than BHT	Fatemi et al. (2014)
*Salvia mirzayanii* Rech.f. & Esfand.	Aerial parts	β-Thujone, 1,8-cineole, camphor	Comparable to trolox	Izadi and Mirazi (2020) Omidpanah et al. (2015)
*Satureja bachtiarica* Bunge.	Aerial parts	ρ-Cymene, γ-terpinene, carvacrol, thymol	Much lower than BHT	Memarzadeh et al. (2020)
*Satureja khuzistanica* Jamzad.	Aerial Parts	Carvacrol, thymol	A bit lower than BHT	Saei-Dehkordi et al. (2012)
*Satureja rechingeri* Jamzad.	Aerial parts	Carvacrol	Lower than equivalent	Alizadeh (2015)
*Thymus daenensis* Celak.	Aerial parts	Thymol, thymoquinone, carvacrol	Comparable to vitamin C	Golkar et al. (2020)
	Aerial parts	Thymol, γ-terpinene, *p*-cymene, carvacrol	A bit lower than BHT	Alavi et al. (2010)
*Thymus kotschyanus* Boiss. & Hohen	Leaves	Carvacrol, β-caryophyllene, γ-terpinene	A bit lower than BHT	Shafaghat et al. (2010) Shafaghat and Shafaghatlonba (2011)
	Aerial parts	γ-Terpinene, thymol, carvacrol	Much lower than BHT	Amiri (2012)
*Zataria multiflora* Boiss.	Aerial parts	Thymol, carvacrol, *p*-cymene, γ-terpinene	Much lower than BHT	Dini et al. (2015); Fatemi et al. (2011)
	Aerial parts	Thymol, carvacrol, *p*-cymene	Lower than BHA and BHT	Hashemi et al. (2011)
*Zhumeria majdae* Rech.f. & Wendelbo	Aerial parts	Linalool, camphor, *trans*-linalool oxide	Much lower than BHT and vitamin C	Saeidi et al. (2019)
*Ziziphora clinopodioides* Lam.	Flowering tops	Pulegone, menthone, limonene	Lower than BHT	Hazrati et al. (2020)
*Rosa damascena* P. Mill.	Flowers	Nonadecane, 9-nonadecane, eicosane	Higher than BHT	Kheirkhahan et al. (2020)

**Table 3.** Antimicrobial activity of Iranian essential oils; part used, major chemical compounds and MIC values.

Scientific names	Part used	Major phytochemical compounds	Inhibited pathogens	MIC values	Ref.
*Bunium persicum* Boiss.	Fruits	γ-Terpinene cuminaldehyde *p*-Cymene	*Staphylococcus aureus* (ATCC 6538)*Escherichia coli* (ATCC 8739)*Candida albicans* (ATCC 10231)	>10 μg/mL >10 μg/mL 2.5–5 μg/mL	Rustaie et al. (2016)
	Fruits	β-Pinene *p*-Cymene γ-Terpinene cumin aldehyde *p*-Mentha-1,3-dien-7-al *p*-Mentha-1 4-Dien-7-al	*Listeria monocytogenes Listeria grayi*	0.351 mg/mL 2.812 mg/mL	Sharafati Chaleshtori et al. (2018)
	Fruits	Cuminaldehyde *p*-Cymene γ-Terpinene safranal	*S. aureus* (ATCC 25923)*Bacillus cereus* (ATCC 11778)*L. monocytogenes* (ATCC 9112)*E. coli* O157:H7 (ATCC700728)*Salmonella enteritidis* (RITCC 1624)	0.75 mg/mL 0.18 mg/mL 0.75 mg/mL 1.50 mg/mL 3 mg/mL	Oroojalian et al. (2010)
*Carum carvi* L.	Seeds	–	*E. coli* (ATCC 25922) *Pseudomonas aeruginosa* (ATCC 27853) *Bacillus subtilis* (ATCC 6633)*S. aureus* (ATCC 29213)	35 ± 1.10 μL/mL 1000 ± 29 μL/mL 9 ± 0.28 μL/mL 16 ± 0.28 μL/mL	Sayhoon et al. (2013)
*Chaerophyllum macropodum* Boiss.	Leaves Flowers	*Trans*-β-Farnesene *Trans*-β-Ocimene β-Pinene limonene spathulenol myrcene	*P. aeruginosa* (ATCC 27853)*E. coli* (ATCC 10536)*Bacillus subtilis* (ATCC 6633)*S. aureus* (ATCC 29737)*Klebsiella pneumoniae* (ATCC 10031)*Staphylococcus epidermidis* (ATCC 12228)*Shigella dysenteriae* (PTCC 1188) *Proteus vulgaris* (PTCC 1182*) Salmonella paratyphi* (ATCC 5702)*C. albicans* (ATCC 10231) *Aspergillus niger* (ATCC 16404)	500 μg/mL 500 μg/mL 250 μg/mL 500 μg/mL 125 and 250 μg/mL 31.3 and 250 μg/mL − 250 μg/mL 125 μg/mL 500 μg/mL –	Ebrahimabadi et al. (2010)
	Aerial parts	Myrcene (*E*)-β-Ocimene terpinolene (*Z*)-β-Ocimene	*A. niger* (ATCC 16888)*Aspergillus oryzae* (ATCC 1011)*Penicillium chrysogenum* (ATCC 10106) *Trichoderma harzianum* (ATCC66765)*Byssochlamys spectabilis* (ATCC 90900)*Paecilomyces variotii* (ATCC 13435)	2500 μg/mL 2500 μg/mL 1250 μg/mL 625 μg/mL 2500 μg/mL 1250 μg/mL	Khajehie et al. (2017)
*Cuminum cyminum* L.	Seeds	β-Pinene γ-Terpinene-7-al γ-Terpinene cumin aldehyde *p*-Cymene	*E. coli* (ATCC 25922) *P. aeruginosa* (ATCC 27853) *B. cereus* (ATCC 9634) *S. aureus* (ATCC 25923)	3.11 ± 0.006 mg/mL 5.2 ± 1.04 mg/mL 3.12 ± 0.00 mg/mL 2.07 ± 0.51 mg/mL	Zolfaghari et al. (2015)
	Seeds	Cumin aldehyde γ-Terpinene *o*-Cymene	*E. coli* O157:H7*L. monocytogenes*	1.93 ± 0.11% 1.13 ± 0.11%	Ekhtelat et al. (2019)
*Ferulago angulata* (Schlecht.) Boiss.	Aerial parts	α-Pinene *Z*-β-Ocimene bornyl acetate *p*-Cymene	*P. aeruginosa* (ATCC 27853)*Shigella flexneri* 2a (LS3)*Micrococcus luteus* (ATCC 10240)*E. faecalis* (ATCC29122)*K. pneumoniae* (ATCC 700603)*Proteus mirabilis* (ATCC 9240)	30–73.3 μg/mL 40–96.6 μg/mL 43.3–80 μg/mL 33.3–80 μg/mL >100 μg/mL 60–96.6 μg/mL	Shahbazi et al. (2016)
	Aerial parts	*cis*-β-Ocimene α-pinene α-phellandrene	*S. aureus E. faecalis E. coli P. aeruginosa*	2 mg/mL 2 mg/mL 4 mg/mL 8 mg/mL	Mumivand et al. (2019)
	Seeds	(*Z*)-β-Ocimene α-pinene *p*-Cymene sabinene β-Phellandrene α-Phellandrene	*Erwinia amylovora Xanthomonas oryzae Pseudomonas syringae Pectobacterium carotovorum Ralstonia solanacearum Bacillus thuringiensis Alternaria alternata Curvularia fallax Macrophomina phaseolina Fusarium oxysporum Cytospora sacchari Colletotrichum trichellum*	12.5 μL/mL 12 μL/mL 17.5 μL/mL 20 μL/mL 20 μL/mL 8 μL/mL 35.40 ± 3.07 to 75.50 ± 4.05 μL/mL 22.73 ± 5.10 to 75.83 ± 4.29 μL/mL 30.64 ± 6.65 to 76.94 ± 4.75 μL/mL 61.80 ± 4.60 to 100.0 ± 0.00 μL/mL 23.90 ± 4.15 to 100.0 ± 0.00 μL/mL 17.60 ± 2.50 to 58.80 ± 3.10 μL/mL	Moghaddam et al. (2018)
	Aerial parts	α-Pinene *cis*-β-Ocimene	*B. cereus L. monocytogenes S. aureus S. typhimurium*	>500 μg/mL 62–500 μg/mL >500 μg/mL >500 μg/mL	Ghasemi Pirbalouti et al. (2016)
	Aerial parts	α-Pinene (*Z*)-Beta-ocimene bornyl acetate γ-Terpinene germacrene D myrcene *p*-Cymene	*S. aureus B. subtilis B. cereus L. monocytogenes S. typhimurium E. coli* O157:H7	50 μg/mL 50 μg/mL 40 μg/mL 40 μg/mL 50 μg/mL 50 μg/mL	Shahbazi et al. (2015)
*Heracleum persicum* Desf.	Seeds	–	*S. aureus* (ATCC 25913)*E. coli* (ATCC 8739)*Salmonella enterica* (PTCC 1709)*Vibrio cholera* (PTCC 1611)*Yersinia enterocolitica* (PTCC 1477)	11% 30% 32% 8% 18%	Shariatifar et al. (2017)
	Aerial parts	Hexyl butanoate octyl isobutyrate octyl 2-methylbuyrate pentylcyclopropane	*L. monocytogenes*	2.5 mg/mL	Ehsani et al. (2019)
	Aerial parts	Hexyl butanoate octyl isobutyrate octyl 2-methylbuyrate, pentylcyclopropane	*L. monocytogenes E. coli*	2.5 mg/mL 5 mg/mL	Rezayan and Ehsani (2015)
*Prangos ferulacea* (L.) Lindl.	Leaves	*p*-Cymene limonene (*E*)-β-ocimene terpinolene 2,3,6-trimethylbenzaldehyde	*B. cereus* (ATCC 11778)*L. innocua* (ATCC 33090)*S. aureus* (ATCC 25923)*E. coli* (ATCC 15224) *S. typhimurium* (ATCC202026) *E. aeruginosa* (ATCC 13048)	6.25 mg/mL 6.25 mg/mL 4.30 mg/mL 12.50 mg/mL 25.00 mg/mL 25.00 mg/mL	Seidi Damyeh et al. (2016)
	Leaves	(*E*)-β-Ocimene *p*-Cymene 2,3,6-trimethylbenzaldehyde limonene terpinolene	*B. cereus* (ATCC 11778) *Listeria innocua* (ATCC 33090) *S. aureus* (ATCC 25923) *E. coli* (ATCC 15224) *S. typhimurium* (ATCC202026) *Enterobacter aerogenes* (ATCC 13048)	6.25 mg/mL 12.5 mg/mL 4.3 and 5.5 mg/mL 12.5 and 25 mg/mL 25 mg/mL 25 mg/mL	Seidi Damyeh and Niakousari (2016)
*Achillea millefolium* L.	Aerial parts	Borneol α-Pinene β-Pinene 1,8-Cineole	*S. aureus* (ATCC 25923)*S. enteritidis* (ATCC 4933)*E. coli* (ATCC 25922)*Penicillium glaucum* (ATCC 9849P) *Saccharomyces cerevisiae* (ATCC 60782)	4.5 and 6.53 mg/mL 7.2 mg/mL 7.2 mg/mL 0.45 and 1.67 mg/mL 0.45 and 2.41 mg/mL	Ahmadi-Dastgerdi et al. (2017)
*Seriphidium kermanense* (D. Podl.) Y. R. Ling [syn: *Artemisia kermanensis* Podl.]	Aerial parts	α-Thujone camphor β-Thujone *p*-Mentha-1 5-Dien-8-ol	*P. aeruginosa S. aureus K. pneumonia*	62 μg/mL 48 μg/mL 54 μg/mL	Gavanji et al. (2014)
	Aerial parts	Isoborneol camphor carvotanacetone	*B. cereus* (ATCC 6633)*B. subtilis* (ATCC 9372) *Enterobacter* spp*E. coli* (ATCC 25922)*Citrobacter* spp*K. pneumoniae* (ATCC 27736)*P. aeruginosa* (ATCC 27852)*S. aureus* (ATCC 25923)*A. niger* (ATCC 9142) *C. albicans* (ATCC 6258)	5.0 mg/mL 1.25 mg/mL 2.5 mg/mL 2.5 mg/mL − 2.5 mg/mL 1.25 mg/mL 1.25 mg/mL 2.5 mg/mL –	Kazemi et al. (2011)
*Dracocephalum kotschyi* Boiss.	Aerial parts	α-Pinene geranial geranyl acetate geraniol	*S. aureus* (ATCC 12600)*S. epidermidis* (PTCC 1435)*Streptococcus agalactiae* (PTCC 1768)*Streptococcus mutans* (PTCC 1683)*E. faecalis* (ATCC 29219)*L. monocytogenes* (ATCC13932)*E. coli* (ATCC11775)*S. typhi* (PTCC 1609)*S. paratyphi A* (PTCC 1230)*S. enterica* (PTCC 1709)*P. aeruginosa* (ATCC 27853)*K. pneumoniae* (ATCC 700603)	160 μg/mL 80 μg/mL 80 μg/mL 80 μg/mL 640 μg/mL 160 μg/mL 640 μg/mL 80 μg/mL 160 μg/mL 160 μg/mL 320 μg/mL 320 μg/mL	Ashrafi et al. (2017)
	Aerial parts	Limonene	*S. aureus* (ATCC 25923)*E. coli* (ATCC 25922)	2–4 mg/mL 4–16 mg/mL	Moridi Farimani et al. (2017)
	Aerial parts	α-Pinene limonene cyclohexylallene neral	*S. epidermidis* (ATCC 12228)*S. aureus* (ATCC 29737)*B. subtilis* (ATCC 6633)*K. pneumonia* (ATCC 10031)*S. dysenteriae* (PTCC 1188)*P. aeruginosa* (ATCC135 27853)*S. paratyphi-A serotype* (ATCC 5702)*P. vulgaris* (PTCC 1182)*E. coli* (ATCC 10536)*A. niger* (ATCC 16404)*Aspergillus brasiliensis* (PTCC 5011)*C. albicans* (ATCC 10231)	125–250 μg/mL 125–500 μg/mL 31.25–125 μg/mL 125 μg/mL 125–500 μg/mL 500–1000 μg/mL 125–250 μg/mL 250–500 μg/mL 1000 μg/mL 500–2000 μg/mL 500–2000 μg/mL 62.5 μg/mL	Ghavam et al. (2021)
	Flowering fragments	α-Pinene geraniol geranial limonene	*K. pneumoniae*	1250–5000 μg/mL	Shakib et al. (2018)
	Aerial parts	Limonene perilla aldehyde	*S. aureus* (ATCC 6538)*S. epidermidis* (ATCC 12228)*E. coli* (ATCC 8739)*P. aeruginosa* (ATCC 9027)	200 μg/mL 200 μg/mL 500 μg/mL 900 μg/mL	Khodaei et al. (2018)
*Hymenocrater calycinus* (Boiss.) Benth.	Aerial parts	1,8-Cineole β-Pinene α-Pinene	*B. subtilis* (PTCC 1023)*S. aureus* (PTCC 1112)*E. coli* (PTCC 1330)*S. typhi* (PTCC 1639)*P. aeruginosa* (PTCC 1074)*A. niger* (PTCC5011)*C. albicans* (PTCC 5027)	1.6 mg/mL 0.8 mg/mL 1.6 mg/mL 1.6 mg/mL – – –	Morteza-Semnani et al. (2012)
*Hymenocrater longiflorus* Benth.	Stems	α-Pinene 1,8-Cineole linalool *p*-Menth-1-en-8-ol β-Bourbonene *trans*-caryophyllene	*E. faecalis* (ATCC 29122) *S. aureus* (ATCC 11522)*K. pneumonia* (ATCC 13183)*P. aeruginosa* (ATCC 27853) *S. flexneri* 2a (LS3)*Salmonella typhimurium* (ATCC 19430)*E. coli* (ATCC 11522) *A. niger C. albicans*	>480 μg/mL 120 μg/mL >480 μg/mL >480 μg/mL >480 μg/mL >480 μg/mL >480 μg/mL 480 μg/mL 240 μg/mL	Ahmadi et al. (2010)
*Mentha piperita* L.	Aerial parts	Menthol menthyl acetate	*C. albicans Candida tropicalis* *Candida krusei Candida glabrata Candida dubliniensis Candida parapsilosis Cryptococcus neoformans A. flavus* (ATCC 64025)*Aspergillus fumigatus* (ATCC 14110)*Aspergillus fumigates* (CBS 144.89)*Aspergillus clavatus* (CBS 514.65)*A. oryzae* (CBS 818.72)	1.5 μL/mL 1.0 μL/mL 0.5 μL/mL 1.2 μL/mL 2.4 μL/mL 4.0 μL/mL 4.0 μL/mL 4.0 μL/mL 0.5 μL/mL 2.0 μL/mL 0.5 μL/mL 2.0 μL/mL	Saharkhiz et al. (2012)
	Aerial parts	Menthofuran menthol 1s-neomenthyl acetate	*S. epidermidis B. subtilis S. aureus S. dysenteriae K. pneumonia*	31.25 μg/mL 31.25 μg/mL 62.50 μg/mL 62.50 μg/mL 31.25 μg/mL	Yazdani et al. (2019)
*Salvia mirzayanii* Rech.f. & Esfand.	Aerial parts	Spathulenol linalool 1,8-Cineole α-Cadinol linalyl acetate terpinenyl acetate cubenol aromadendrone thymol	*B. subtilis Bacillus pumilus E. faecalis S. aureus S. epidermidis E. coli P. aeruginosa K. pneumoniae*	1.87 mg/mL 1.87 mg/mL − 7.5 mg/mL 7.5 mg/mL 15 mg/mL – –	Armana et al. (2012)
	Aerial parts	α-Terpinyl acetate geranial linalool 1,8-Cineole	*S. aureus E. coli C. albicans*	40.6 ± 2.1 to 62.5 ± 2.4 μg/mL 22.4 ± 1.8 to 33.6 ± 1.2 μg/mL 28.5 ± 1.7 to 42.4 ± 1.8 μg/mL	Ghasemi et al. (2020)
	Aerial parts	Cineol linalyl acetate	*Streptococcus mutants* (ATCC 35668)*Streptococcus sanguinis* (ATCC 10556)*Streptococcus salivarius* (ATCC 9222)*Streptococcus sobrinus* (ATCC 27607)*E. faecalis* (ATCC11700)*S. aureus* (ATCC 25923, 29213 and ATCC 700698)*Candida albicans* (ATCC 10261)*C. dubliniensis* (CBS 8501)*Candida tropicalis* (ATCC750) *Candida krusei* (ATCC 6258)*Candida glabrata* (ATCC 90030)	0.062–0.125 μL/mL 0.125 μL/mL 0.5 μL/mL 0.125 μL/mL 0.25 μL/mL 0.062–0.125 μL/mL 1 μL/mL 0.5 μL/mL 0.25 μL/mL 1 μL/mL 0.25 μL/mL	Zomorodian et al. (2015)
	Leaves	1,8-Cineole linalool acetate α-Terpinyl acetate	*C. albicans* (ATCC) *C. tropicalis* (ATCC 750)*C. krusei* (ATCC 6258)*C. glabrata* (ATCC 863, 2192, 2175, 6144)*C. dubliniensis* (CBS 8501, ATCC 8500)*C. parapsilosis* (ATCC 4344)*S. aureus* (ATCC 25923)*E. faecalis* (ATCC11700)*E. coli* (ATCC 43894)*S. mutans* (ATCC 35668)*Streptococcus pneumonia* (ATCC33400) *Streptococcus pyogenes* (ATCC 8668)*S. enterica* (ATCC 14028)*S. flexneri* (NCTC 8516)*P. aeruginosa*	0.5–2 μL/mL 0.25 μL/mL 1 μL/mL 0.03–0.5 μL/mL 0.03–0.12 μL/mL 0.12–0.5 μL/mL 0.12–0.32 μL/mL 0.35–0.37 μL/mL 16–64 μL/mL 0.06 μL/mL >0.031 μL/mL >0.031 μL/mL >128 μL/mL 16 μL/mL >128 μL/mL	Zomorodian et al. (2017)
*Satureja bachtiarica* Bunge	Aerial parts	Carvacrol thymol	*Helicobacter pylori*	0.035 ± 0.13 μL/mL	Falsafi et al. (2015)
	Aerial parts	*p*-Cymene thymol carvacrol	*P. aeruginosa*	31 μg/mL	Ghasemi Pirbalouti and Dadfar (2013)
	Aerial parts	Thymol, carvacrol	*S. aureus* (PTCC1431)*B. cereus* (PTCC 1015)*E. coli* (PTCC1399)*P. aeruginosa* (PTCC1430)	1.0 mg/mL 1.0 mg/mL 0.5 mg/mL >64 mg/mL	Hadian et al. (2012)
	Aerial parts	Carvacrol thymol γ-Terpinene *p*-Cymene	*B. cereus S. aureus S. agalactiae L. monocytogenes P. vulgaris S. typhimurium*	62.5–500 μg/mL 62.5–500 μg/mL 31.2–125 μg/mL 16–250 μg/mL 31.2–500 μg/mL 16–250 μg/mL	Ghasemi Pirbalouti et al. (2017)
	Aerial parts	Carvacrol thymol *o*-Cymene	*L. monocytogenes*	5 mg/mL	Fathi-moghaddam et al. (2020)
	Aerial parts	Carvacrol *p*-Cymene thymol	*S. aureus*	62 μg/mL	Ghasemi Pirbalouti et al. (2014)
*Satureja khuzistanica* Jamzad	Aerial parts	Carvacrol	*E. coli*	0.14 ± 0.08 μL/mL	Mahboubi and Kazempour (2016)
	Aerial parts	Thymol carvacrol borneol linalool	*Lactobacillus plantarum* LU5*S. flexneri E. coli*	12.5 μg/mL 3.125 μg/mL 12.5 μg/mL	Hashemi and Khodaei (2020)
	Aerial parts	Carvacrol γ-Terpinene *p*-Cymene	*S. aureus C. albicans*	0.5 μL/mL 0.2 μL/mL	Golparvar et al. (2018)
	Aerial parts	Carvacrol	*S. aureus* (ATCC 25923) *S.* MRSA (ATCC H041940150) *P. aeruginosa* (ATCC 27853) *L. monocytogenes* (ATCC 35152)	0.16–7.8 mg/mL 0.125–0.625 mg/mL 20–80 mg/mL 0.31–1.25 mg/mL	Rashidipour et al. (2016)
	Aerial parts Aerial parts	Carvacrol thymol carvacrol	*S. aureus* (ATCC 2228) *L. monocytogenes* (ATCC 19118) *B. cereus* (ATCC11778)*B. subtilis* (ATCC 12711) *S. pneumoniae* (ATCC 33400)*E. coli* O157:H7 (ATCC 43895)*S. typhimurium* (ATCC 14028)*P. aeruginosa* (ATCC 9027) *K. pneumoniae* (ATCC 10031)*C. albicans* (ATCC 10231)*C. tropicalis* (ATCC 13801)*Rhodotorula mucilaginosa* (ATCC 2503)*Rhodotorula rubra* (PTCC 5076)	360 μg/mL 270 μg/mL 540 μg/mL 540 μg/mL 540 μg/mL 720 μg/mL 360 μg/mL 1080 μg/mL 540 μg/mL 180 μg/mL 270 μg/mL 90 μg/mL 135 μg/mL	Saei-Dehkordi et al. (2012)
*Satureja rechingeri* Jamzad	Aerial parts	Carvacrol γ-Terpinene linalool *p*-Cymene thymol	*C. albicans* (ATCC 10231) *S. aureus* (ATCC 6538) *S. epidermidis* (ATCC 1435)*E. coli* (ATCC 25922)	0.19 mg/mL 0.39 mg/mL 0.39 mg/mL 0.19 and 0.39 mg/mL	Alizadeh (2015)
	Aerial parts	Thymol carvacrol	*S. aureus* (PTCC1431)*B. cereus* (PTCC 1015)*E. coli* (PTCC1399)*P. aeruginosa* (PTCC1430)	0.5 mg/mL 0.25 mg/mL 0.25 mg/mL >64 mg/mL	Hadian et al. (2012)
*Thymus daenensis* Celak.	Aerial parts	Thymol terpinene *p*-Cymene carvacrol	*C. albicans* (ATCC 10231)	0.2 μL/mL	Hadipanah and Khorami (2016)
	Aerial parts	Thymol carvacrol	*A. fumigatus A. niger*	0/5 ± 0/05 mg/mL 1 ± 0/1 mg/mL	Mohammadi Gholami et al. (2018)
	Aerial parts	Thymol thymoquinone carvacrol	*E. coli* (ATCC35218) *S. typhimurium* (ATCC14028)*S. aureus* (ATCC29213)*B. cereus* (ATCC14579)	20 μg/mL 20 μg/mL 20 μg/mL 20 μg/mL	Golkar et al. (2020)
	Aerial parts	Thymol carvacrol *p*-Cymene	*B. subtilis* (PTCC-1156)*B. cereus* (PTCC-1247)*S. pyogenes* (PTCC-1447)*M. luteus* (ATCC 10987)*E. faecalis* (PTCC-1195)*S. aureus* (PTCC-1189)*S. typhi* (PTCC-1609)*P. aeruginosa* (PTCC-1181) *E. coli* (PTCC-2922) *Shigella boydii* (PTCC1744)*Enterobacter aerogenes* (PTCC-1221) *Acinetobacter baumannii* (PTCC-4413)*Proteus mirabilis* (ATCC-1287) *Neisseria meningitidis* (PTCC-4578)*K. pneumoniae* (ATCC-1129)	7.5 μg/mL 15 μg/mL 7.5 μg/mL 7.5 μg/mL 3.75 μg/mL 15 μg/mL − 15 μg/mL - − 15 μg/mL 15 μg/mL − 15 μg/mL 15 μg/mL	Alamholo (2020)
*Thymus kotschyanus* Boiss. & Hohen	Aerial parts	Carvacrol 1,8-Cineole thymol borneol *E*-Caryophyllene	*C. albicans* (ATCC 10231 BBL) *S. aureus* (ATCC 6538)*S. epidermidis* (PTCC1435)*B. cereus* (PTCC1247)*E. coli* (PTCC1399)	3.645 μL/mL 1.562 μL/mL 0.097 μL/mL 1.562 μL/mL 6.25 μL/mL	Ahmadi et al. (2015)
	Aerial parts	Carvacrol β-Caryophyllene γ-Terpinene α-Phellandrene *p*-Cymene thymol	*S. faecalis E. coli S. typhi S. aureus C. albicans*	– 50 μg/mL − 100 μg/mL –	Asbaghian et al. (2011)
*Zataria multiflora* Boiss.	Aerial parts	Thymol carvacrol *p*-Cymene γ-Terpinene	*E. coli* (ATCC 25922) *P. aeruginosa* (ATCC 27853)*B. cereus* (ATCC9634)*S. aureus* (ATCC 25923)	1.3 ± 0.4 mg/mL 2.6 ± 0.9 mg/mL 1.3 ± 0.4 mg/mL 1.2 ± 0.7 mg/mL	Fatemi et al. (2015)
	Aerial parts	Carvacrol thymol *p*-Cymene	*E. coli* O157:H7	0.015–0.03% (v/v)	Khatibi et al. (2017)
	Aerial parts	Thymol *p*-Cymene γ-Terpinene	*E. coli* O157: H7 (ATCC 10536)*S. typhimurium* (ATCC 14028) *L. monocytogenes* (ATCC19118)*B. cereus* (ATCC 11778) *S. aureus* (ATCC 6538)	500 ppm 500 ppm 500 ppm 250 ppm 500 ppm	Javan (2016)
	Aerial parts	–	*L. monocytogenes* (ATCC1911)	1.2–9.5 μg/mL	Rahnama et al. (2012)
	–	–	*Aspergillus* spp.*Rhizoctonia solani Rhizopus stolonifer*	200 ppm 300 ppm 200 ppm	Nasseri et al. (2016)
	–	–	*L. monocytogenes S. typhimurium*	2500 μg/mL 5000 μg/mL	Shahabi et al. (2017)
	Aerial parts	Carvacrol thymol	*L. monocytogenes* (ATCC 19118)	78.10–312.50 μg/mL	Raeisi et al. (2016)
	Aerial parts	–	*S. aureus* (ATCC 25923) *S. aureus* MRSA (ATCC 33591) *S. epidermidis* (ATCC 14990) *P. aeruginosa* (ATCC 27853)	3.125 mg/mL 3.125 mg/mL 6.25 mg/mL 12.5 mg/mL	Sheikholeslami et al. (2016)
	Aerial parts	Carvacrol γ-Terpinene α-Pinene	*Lactococcus garvieae*	7.8 μg/mL	Mahmoodi et al. (2012)
	Aerial parts	Thymol carvacrol *p*-Cymene	*P. aeruginosa* (ATCC 27853)	2 μL/mL	Mahboubi et al. (2017)
	Aerial parts	Thymol carvacrol *p*-Cymene	*A. flavus*	100 ppm	Rahimi et al. (2019)
	Leaves	Thymol carvacrol *p*-Cymene	*P. aeruginosa* (ATCC 9027)*S. typhi* (ATCC 10031)*K. pneumonia* (PTCC 1053) *E. coli* (ATCC 8739) *S. aureus* (PTCC 1112)*S. epidermidis* (ATCC 12228) *B. subtilis* (ATCC 6633)*A. niger* (PTCC 5010) *C. albicans* (PTCC 5027)	– 2.65 ± 0.7 μg/mL 2.85 ± 0.5 μg/mL 2.72 ± 0.8 μg/mL 3.02 ± 0.8 μg/mL 3.53 ± 1 μg/mL 3.65 ± 0.9 μg/mL 2.2 ± 0.5 μg/mL 2.8 ± 0.8 μg/mL	Mahammadi Purfard and Kavoosi (2012)
	Leaves	Thymol carvacrol linalool	*S. typhimurium* (ATCC 14028) *L. monocytogenes* (ATCC 19117)	0.625 mg/mL 1.25 mg/mL	Mojaddar Langroodi et al. (2019)
	Aerial parts	Thymol carvacrol *p*-Cymene	*B. cereus* (PTCC 1023)*P. aeruginosa* (PTCC 1310) *P. vulgaris* (PTCC1449)*S. cerevisiae* (PTCC24860) *C. utilis* (PTCC5052)*P. digitatum* (ATCC 201167) *A. niger* (PTCC 5011)	50 μg/mL 25 μg/mL 25 μg/mL 200 μg/mL 100 μg/mL 200 μg/mL 200 μg/mL	Avaei et al. (2015)
*Zhumeria majdae* Rech.f. & Wendelbo	Aerial parts	Linalool camphor limonene	*B. cereus B. thuringiensis E. faecalis S. epidermidis S. typhimurium Campylobacter jejuni K. pneumoniae P. fluorescens*	0.93 mg/mL 0.93 mg/mL 7.5 mg/mL 5 mg/mL 10 mg/mL 15 mg/mL 10 mg/mL 15 mg/mL	Mirzakhani et al. (2018)
*Ziziphora clinopodioides* Lam.	Flowering tops	Pulegone menthone limonene	*B. subtilis* (ATCC465)*B. pumilus* (PTCC 1274) *S. aureus* (ATCC 25923)*E. coli* (ATCC 25922)*K. pneumoniae* (ATCC 10031)*S. epidermidis* (ATCC9763)*B. cereus* (PTCC 1015)	7.5 mg/mL 7.5 mg/mL 7.5 mg/mL 15 mg/mL 15 mg/mL >15 mg/mL 7.5 mg/mL	Hazrati et al. (2020)
		*p*-Peritone pulegone carvacrol	*Aspergillus parasiticus*	0.37 ± 0.1 mg/mL	Khosravi et al. (2011)
	Leaves	Carvacrol thymol *p*-Cymene γ-Terpinene	*S. aureus* *B. subtilis* *B. cereus* *L. monocytogenes* *S. typhimurium* *E. coli* O157:H7	0.0025 μL/mL 0.0012 μL/mL 0.0012 μL/mL 0.0012 μL/mL 0.0025 μL/mL 0.0025 μL/mL	Shahbazi (2015)
	Fresh leaves stem flowers	Carvacrol thymol γ-Terpinene *p*-Cymene	*S. aureus* (ATCC 6538) *B. subtilis* (ATCC 6633) *B. cereus* (ATCC 11774) *L. monocytogenes* (ATCC 19118) *S. typhimurium* (ATCC 14028) *E. coli* O157:H7 (ATCC 10536)	0.03 ± 0.00 to 0.04 ± 0.00% 0.03 ± 0.00 to 0.04 ± 0.00% 0.04 ± 0.00 to 0.05 ± 0.00% 0.03 ± 0.00 to 0.04 ± 0.00% 0.04 ± 0.00 to 0.05 ± 0.00% 0.04 ± 0.00 to 0.05 ± 0.00%	Shahbazi (2017)
*Rosa damascena* P. Mill.	Flowers	β-Citronellol geraniol	*S. aureus* (ATCC 25923)*Staphylococcus saprophyticus* (ATCC 15305)*S. epidermidis* (ATCC 14490)*B. cereus* (ATCC 1247) *B. subtilis* (ATCC6051) *S. pyogenes* (ATCC 8668)*S. agalactiae E. faecalis* (ATCC 29212) *Enterococcus faecium* (ATCC 25778)*S. sanguis* (ATCC 10556)*S. salivarius* (ATCC 9222)*K. pneumonia* (ATCC 10031)*E. coli* (ATCC 8739)*S. typhimurium* (ATCC 14028)*P. aeruginosa* (ATCC 9027)*P. vulgaris* (RI 231)*E. aerogenes* (NCTC 10009)*S. dysenteriae* (RI 366)*S. flexneri* (NCTC 8516)*Serratia marcescens* (ATCC 13880)*C. albicans* (ATCC10231)*A. flavus A. niger* (ATCC 16404)*A. parasiticus* (ATCC 15517)	1 μg/mL 0.5 μg/mL 0.5 μg/mL 0.5 μg/mL 0.5 μg/mL 0.25 μg/mL 1 μg/mL 1 μg/mL 1 μg/mL 1 μg/mL 1 μg/mL 0.125 μg/mL 1 μg/mL 1 μg/mL 1 μg/mL 1 μg/mL 0.125 μg/mL 1 μg/mL 0.5 μg/mL 0.5 μg/mL 1 μg/mL 0.5 μg/mL 0.125 μg/mL 0.25 μg/mL	Mahboubi et al. (2011)
	Petals	Nonadecane 9-Nonadecane eicosane	*S. aureus E. coli S. typhi*	250 μL/mL 500 μL/mL 1000 μL/mL	Kheirkhahan et al. (2020)
	–	Nonadecane heneicosane β-Citronellol	*C. albicans C. tropicalis C. krusei C. glabrata C. dubliniensis A. flavus A. fumigatus A. clavatus* (CBS 514.65) *A. oryzae* (CBS 818.72)*Cryptococcus neoformans* (ATCC2406)*S. aureus* (ATCC29213) *E. faecalis* (ATCC 11700)*E. coli* (ATCC 25922)*P. aeruginosa* (ATCC27853)	2 μL/mL 8 μL/mL 8 μL/mL 1 μL/mL 1 μL/mL 2 μL/mL 0.5 μL/mL 0.5 μL/mL 1 μL/mL 0.25 μL/mL 8 μL/mL >64 μL/mL 16 μL/mL >64 μL/mL	Moein et al. (2017)

The essential oils with highly inhibitory effects against Gram-positive bacteria are highlighted in grey.

## *Bunium persicum* (Boiss.) B. Fedtsch. (Apiaceae)

**Figure 1.**
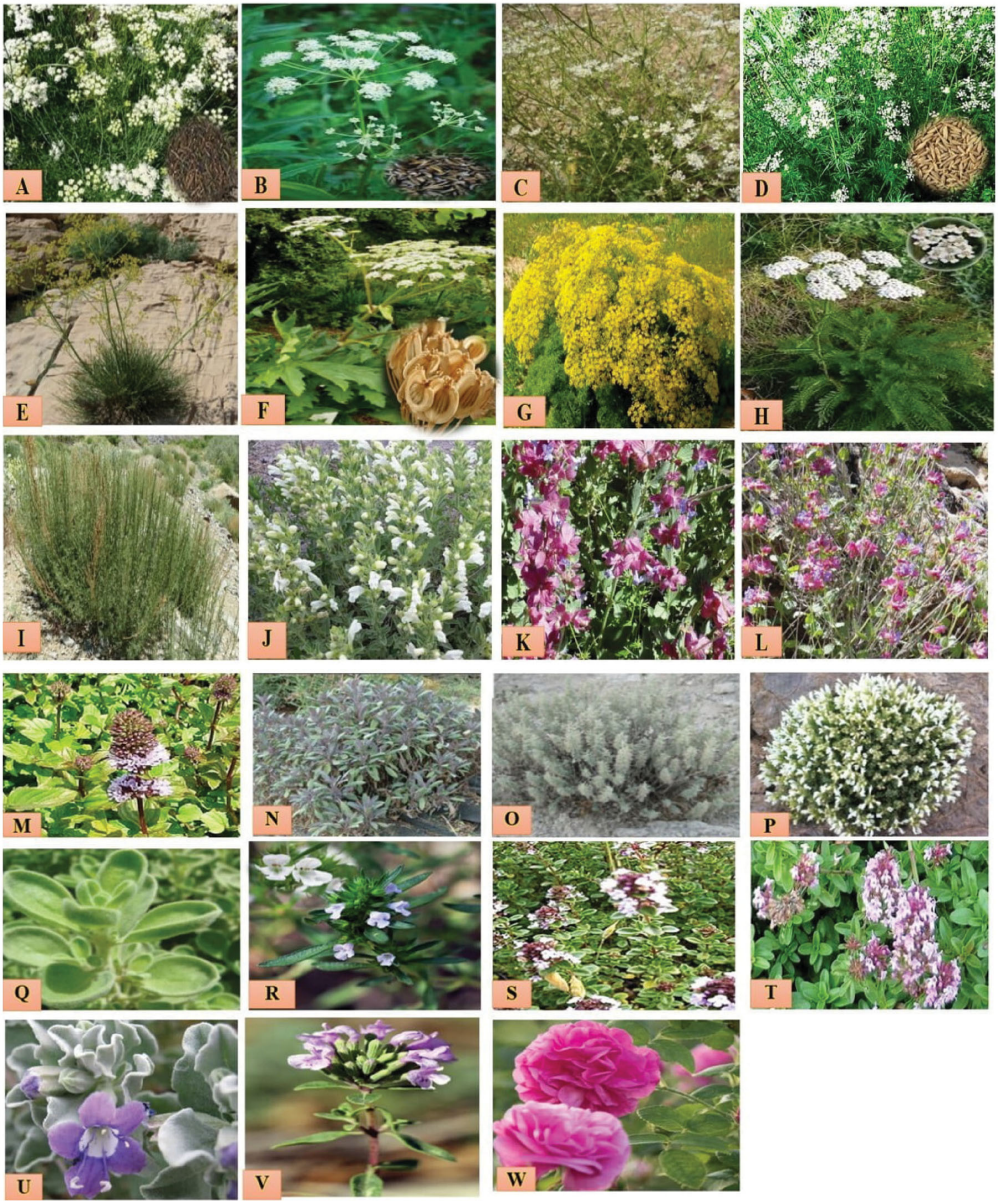
Some Iranian medicinal plants: (A) *B. persicum*, (B) *C. carvi*, (C) *C. macropodum*, (D) *C. cyminum*, (E) *F. angulata*, (F) *H. persicum*, (G) *P. ferulacea*, (H) *A. millefolium*, (I) *S. kermanense*, (J) *D. kotschyi*, (K) *H. calycinus*, (L) *H. longiflorus*, (M) *M. piperita*, (N) *S. mirzayanii*, (O) *S. bachtiarica*, (P) *S. khuzistanica*, (Q) *S. rechingeri*, (R) *T. daenensis*, (S) *T. kotschyanus*, (T) *Z. multiflora*, (U) *Z. majdae*, (V) *Z. clinopodioides* and (W) *R. damascena*.

*B. persicum* (Figure 1(A)) is traditionally used to improve digestion, besides a flavouring and carminative agent and to treat varied ailments, viz., toothache, stomach-ache and dyspnoea (Pezhmanmehr et al. 2009; Amiri et al. 2012). The essential oil of *B*. *persicum* exhibited significant antioxidant activity using DPPH and β-carotene-linoleic acid (Nickavar et al. 2014) methods in presence of major compounds: β-pinene, p-cymene, cumin aldehyde and γ-terpinene. In previous studies, the antioxidant capacity of *B. persicum* essential oil isolated by two different methods: microwave (MAE) and hydro-distillation (HD) techniques were compared and no significant difference in the antioxidant activities was reported. Consequently, MAE method was recommended due to shorter extraction time and faster energy transfer (Mazidi et al. 2012; Majidi et al. 2020). The DPPH and ABTS radical scavenging assays results indicated that the antioxidant activity of *Zataria multiflora* Boiss. (Lamiaceae) essential oil was greater than *B. persicum* essential oil (Aminzare et al. 2017). The antimicrobial properties of *B. persicum* essential oil with the main chemical compounds: cumin aldehyde, γ-terpinene, β-pinene and *p*-cymene against *Listeria monocytogenes*, as multidrug-resistant bacteria, have been confirmed (Sharafati Chaleshtori et al. 2018). Likewise, *B. persicum* essential oil inhibited the growth of *Candida albicans*, with considerable antibacterial potential against *Staphylococcus aureus* and *Escherichia coli* (Rustaie et al. 2016). Additionally, the synergistic antibacterial activity of *B. persicum* and *Cuminum cyminum* L. (Apiaceae) essential oils against Gram-positive bacteria (*S. aureus*, *B. cereus* and *L. monocytogenes*) has been recorded (Oroojalian et al. 2010).

## *Carum carvi* L. (Apiaceae)

Caraway seeds (*C. carvi*) (Figure 1(B)) are used medicinally as a laxative, carminative, appetite stimulant, besides increasing lactation in pregnant women and alleviating menstrual pain (Haidari et al. 2011; Keshavarz et al. 2012). The *in vitro* antioxidant property of *C. carvi* essential oil measured by β-carotene bleaching and DPPH assays was reported by Fatemi et al. (2011). Moreover, other studies determined the antioxidant activity of *C. carvi* essential oil on liver and lung tissue changes histopathologically and indicated that *C. carvi* essential oil retained the balance via oxidants and antioxidants (Fatemi et al. 2010; Dadkhah et al. 2011, 2018). In a study on antibacterial activity of caraway essential oil, the Gram-positive bacteria; *Bacillus subtilis* and *S. aureus* exhibited more sensitivity in relation to Gram-negative pathogens; *E. coli* and *Pseudomonas aeruginosa* (Sayhoon et al. 2013).

## *Chaerophyllum macropodum* Boiss. (Apiaceae)

*C. macropodum* (Figure 1(C)) is distributed in Iran and Turkey, while other species are detectable in other areas particularly Europe and central Asia (Mozafarian 1996). This plant is not only used in traditional healing practices to treat cold and stomach, but also in culinary (Jahantab et al. 2018; Moazzami Farida et al. 2018). The antioxidant activity of essential oil from *C. macropodum* aerial parts was determined by DPPH radical scavenging and β-carotene bleaching tests. The results indicated that essential oil containing the most prominent bioactive compounds: *trans*-ocimene, *cis*-ocimene and γ-terpinene possessed low antioxidant activity as compared to BHT (Haghi et al. 2010). Additionally, the chemical composition and antioxidant properties of *C. macropodum* essential oil isolated by HD and microwave-assisted hydrodistillation (MAHD) methods were measured and compared. The main constituents of both essential oils obtained by HD and MAHD, were (*E, Z*)-β-ocimene, myrcene and terpinolene, respectively. There was no significant difference in the antioxidant activity of both essential oils (Khajehie et al. 2017). Moreover, MIC concentrations of essential oils obtained from *C. macropodum* leaves and flowers were evaluated against 12 bacterial strains using the micro-well dilution assay. Thirty constituents were identified and their main classes were oxygenated, non-oxygenated monoterpenes and sesquiterpenes (*trans*-β-farnesene and *trans*-β-ocimene). *Salmonella paratyphi*-A serotype, *Proteus vulgaris*, *Staphylococcus epidermidis* and *Klebsiella pneumonia* were the most susceptible species with MIC ranging from 125 to 250 μg/mL (Ebrahimabadi et al. 2010). Khajehie et al. (2017) evaluated the antifungal activities of *C. macropodum* aerial parts essential oil through HD and MAHD techniques by MIC or minimum fungicidal concentrations (MFCs) methods and reported that MAHD had no adverse effects on inhibitory effects of the essential oil, besides *Trichoderma harzianum* was the most sensitive microorganism with MIC of 625 μg/mL.

## *Cuminum cyminum* L. (Apiaceae)

*C. cyminum* (Figure 1(D)), or cumin seeds, is not only the main ingredient of different traditional cuisines, but also has been widely used to cure varied ailments; gastrointestinal diseases, tooth decay, cough, epilepsy, diabetes and aches (Gachkar et al. 2007; Johri 2011; Srinivasan 2018). The essential oil of *C. cyminum* seeds rich in β-pinene, γ-terpinene-7-al and γ-terpinene have considerable radical-scavenging and antioxidant activities that are comparable with Trolox and BHT (Fatemi et al. 2013). Likewise, Ladan Moghadam (2016), showed that the antioxidant activity of *C. cyminum* essential oil was even higher than trolox. Moreover, Zolfaghari et al. (2015), studied *in vitro* and *in vivo* antimicrobial potentiality of cumin seeds essential oil against Gram-positive and negative bacterial strains and revealed that *B. cereus* (MIC = 2.07 ± 0.51 mg/mL) was the most species. Later, the combinations of *C. cyminum* essential oil and standard antibiotics (sodium benzoate) were screened to determine the presence of any synergistic activities. The results demonstrated that the antimicrobial activity of *C. cyminum* essential oil preservatives, when used in combination with other preservatives, was higher. Moreover, the Gram-positive bacteria (MIC = 1.13 ± 0.11%) were more sensitive to *C. cyminum* essential oil than Gram-negative bacteria (MIC = 1.93 ± 0.11%) (Ekhtelat et al. 2019). Similarly, Tavakoli et al. (2015) indicated good antimicrobial activity of *C. cyminum* essential oil combined with nisin (a preservative agent) against *Salmonella typhimurium* growth at 10 °C and *S. aureus* growth at 10 °C and 25 °C, respectively, in BHI broth during study period of 43 days.

## *Ferulago angulata* (Schltdl.) Boiss. (Apiaceae)

Chavil (*F. angulata*) (Figure 1(E)) has been traditionally used as an antiseptic, air freshener and spice in Iranian cuisine (Ghasemi et al. 2013; Bagherifar et al. 2019). The antioxidant capacity of *F. angulata* essential oil collected from diverse Iran provinces was compared. In DPPH assay, the strongest antioxidant effects were found in Chavil collected from Lorestan Province (IC_50_=11.70 ± 0.217 mg/mL) attributed to its main compounds of α-pinene and *Z*-β-ocimene (Shahbazi et al. 2016). Chavil essential oil (α-pinene and *cis*-β-ocimene) from the southwestern regions of Iran also presented an excellent antioxidant activity comparable with BHT (Ghasemi Pirbalouti et al. 2016). The phytochemical composition and antibacterial activity of *F. angulata* essential oil collected from different parts of Iran were assessed by agar dilution and disc diffusion methods. The results showed that the essential oil from *F. angulata* grown in Kurdestan Province containing high amount of α-pinene and *Z*-β-ocimene exhibited highest antibacterial activity against all the tested bacteria particularly *E. faecalis* (MIC and MBC = 33.3 and 40 μg/mL) (Shahbazi et al. 2016). Mumivand et al. (2019) reported that Gram-negative pathogens (*E. coli* and *P. aeruginosa*) were more resistant to essential oil of *F. angulata* aerial parts. Likewise, Moghaddam et al. (2018) studied the antibacterial and antifungal activities of essential oil from *F. angulata* seeds against six bacterial and fungal species and revealed that *Bacillus thuringiensis*, *Fusarium oxysporum* and *Colletotrichum trichellum* were sensitive to essential oil containing high content of *cis*-β-ocimene, α-pinene and α-phellandrene. Similarly, the findings of Ghasemi Pirbalouti et al. (2016) indicated that Chavil essential oil extracted from *F. angulata* had very strong activity against *L. monocytogenes* (Gram-positive bacterium). Similarly, Shahbazi et al. (2015) reported that the two Gram-positive bacteria (*L. monocytogenes* and *B. cereus*) were more sensitive to *F. angulata* essential oil.

## *Heracleum persicum* Desf. (Apiaceae)

Golpar (*H. persicum*) (Figure 1(F)) distributed in Alborz regions has been widely used in traditional medicine and food in different parts of Iran and Middle Eastern countries (Amin 1991; Kousha and Bayat 2012; Roshanaei et al. 2017). In traditional Iranian medicine, fruits and stems of this plant are used as a spice, in pickling (Shariatifar et al. 2017), and as an analgesic, antiseptic, anti-flatulence and digestive aid, as well as remedy for stomach pains and infections (Hemati et al. 2010; Bahadori et al. 2016). In the comparative antioxidant activities of 10 selected herbs via DPPH method, *H. persicum* extract did not show a significant result (Dehghan et al. 2016), contrastingly, comparative study on antioxidant activity of essential oils from four *Heracleum* species; *H. pastinacifolium* C. Koch and *H. persicum* essential oils showed the highest activities which could probably be due to the presence of myristicin and (*E*)-anethole (Firuzi et al. 2010). The data (Shariatifar et al. 2017) obtained from disc diffusion and broth micro-dilution methods demonstrated a notable antimicrobial activity of *H. persicum* essential oil against the selected bacterial strains; *S. aureus* (MIC = 11%), *Salmonella enterica* (MIC = 32%), *E. coli* (MIC = 30%), *Vibrio cholerae* (MIC = 8%) and *Yersinia enterocolitica* (MIC = 18%). Rezayan and Ehsani (2015) reported that the antibacterial effects of *H. persicum* essential oil with principal compounds; hexyl butanoate, octyl isobutyrate, octyl 2-methylbuyrate and pentylcyclopropane, were more significant on *L. monocytogenes* (PTCC 1165) as a Gram-positive bacterium. The highest antimicrobial potentials were reported for essential oil of *H. persicum* on *B. subtilis* (Noudeh et al. 2010). In a study by Ehsani et al. (2019) evaluated *H. persicum* essential oil, nisin and *Lactobacillus acidophilus* (as a probiotic agent) to inhibit the growth of *L. monocytogenes* and reported that a combined formulation containing low concentration of *H. persicum* essential oil, nisin and probiotic agent signified a synergistic effect.

## *Prangos ferulacea* (L.) Lindl. (Apiaceae)

*P. ferulacea* (Figure 1(G)) (Djashir) is used to flavour foods, for medical preparations, and animal fodder. In addition, Djashir is a laxative, wound healing, antihypertensive and carminative agent (Yousefi et al. 2017; Mottaghipisheh et al. 2020). Bazdar et al. (2018) evaluated the antioxidant potential of essential oil and extract from *P. ferulacea* flowers and leaves against DPPH radicals and reported that the hydro alcoholic flowers (IC_50_=8.01 ± 0.60) containing the highest number of flavonoids showed the highest antioxidant activities compared to Djashir essential oil (IC_50_=23.90 ± 2.59 and 22.99 ± 2.13, respectively). A comparative study (Seidi Damyeh et al. 2016) assessed the effects of novel ohmic-assisted hydrodistillation (OAHD) on chemical compositions, besides antioxidant and antibacterial activities of essential oil from *P. ferulacea* leaves and demonstrated a significant difference in the percentage of chemical compositions percent between HD and OAHD, but antioxidant effects (IC_50_=488.14 and 570.52 μg/mL, respectively) were less remarkable than BHT (IC_50_=17.34 μg/mL). OAHD method influence on antibacterial efficacy of *P. ferulacea* essential oil, *B. cereus*, *Listeria innocua*, *S. aureus*, *E. coli*, *S. typhimurium* and *Enterobacter aerogenes* was studied. The essential oil extracted by HD constituting mainly (E)-β-ocimene, p-cymene, 2,3,6-trimethylbenzaldehyde, germacrene D and terpinolene showed better antimicrobial activity, particularly against *S. aureus.* These researchers also indicated that sonication prior to extraction had no significant efficacy on antibacterial effects and chemical compounds of *P. ferulacea* essential oils, and the most sensitive and resistant bacterial species were *S. aureus* and *S. typhimurium*, respectively. Therefore, the ultrasonic pre-treatment of plants prior to extraction could be desirable to minimize the extraction times (Seidi Damyeh and Niakousari 2016).

## *Achillea millefolium* L. (Asteraceae)

*A. millefolium* (Boomadaran) (Figure 1(H)) is an herbaceous flowering plant with several traditional uses, viz., anti-infections, antihemorrhage, anti-inflammation and antidiabetic (Mirdeilami et al. 2011; Mazandarani et al. 2013; Bahmani et al. 2014). *A. millefolium* essential oil exhibited significantly greater radical scavenging activity (IC_50_=23.11 ± 0.04 mg/mL), than trolox (IC_50_=23.51 ± 0.05 mg/mL). β-Carotene bleaching method findings also confirmed its capacity (Sahari Moghadam et al. 2017). Additionally, *A. millefolium*, *A. graveolens* and *Carum copticum* L. (Apiaceae) essential oils were tested for *in vitro* antioxidant activity using DPPH, FRAP and β-carotene bleaching assays. The antioxidant activity of *A. millefolium* essential oil was statistically superior to other tested plants and even trolox. The presence of high levels of phenolic substances, viz., thymol and carvacrol may attribute to the antioxidant properties of *A. millefolium* essential oil (Kazemi 2015). Comparatively, the essential oil of *A. millefolium* leaves had weaker antimicrobial effects than the essential oil from its flowers positively correlating to occurrence of camphor, borneole and α-cadinol. The highest activity of essential oils was observed against *S. aureus*, *Penicillium glaucum* and *S. cerevisiae* (Ahmadi-Dastgerdi et al. 2017).

## *Seriphidium kermanense* (D. Podl.) Y. R. Ling (Asteraceae)

*S. kermanense* [syn. *Artemisia kermanensis* D. Podl.] (Figure 1(I)) is an important herb in the south of Kerman Province, Iran (Mozafarian 1996). In folk medicine, this plant was used for to treat skin disease and high blood pressure (Dolatkhahi et al. 2014). Jamzad (1996) demonstrated that *A. kermanensis* essential oil possessed considerable antioxidant and radical scavenging activities through DPPH and β-carotene-linoleic acid assays (Jamzad 1996). The essential oil was reported to exert antibacterial effects against *B. subtilis*, *P. aeruginosa* and *S. aureus.* The MIC and MBC results demonstrated that *B. subtilis* (MIC = 1.25 mg/mL, MBC = 2.5 mg/mL), *P. aeruginosa* (MIC = 1.25 mg/mL, MBC = 2.5 mg/mL) and *S. aureus* (MIC = 1.25 mg/mL, MBC = 2.5 mg/mL) were the most sensitive microorganisms (Kazemi et al. 2011). Gavanji et al. (2014) evaluated the antimicrobial activity of *A. kermanensis* essential oil against *S. aureus* (ATCC 25923), *P. aeruginosa* (PTCC 1310) and *K. pneumonia* (PTCC 1053), with 54, 62 and 48 μg/mL MIC values, respectively.

## *Dracocephalum kotschyi* Boiss. (Lamiaceae)

*D. kotschyi* (Figure 1(J)) aerial parts are used in traditional medicine to treat stomach, headache, toothache and liver disorders (Heydari et al. 2019; Fallah et al. 2020). The chemical composition and antioxidant activity of *D. kotschyi* essential oil were analysed by DPPH and GC/MS methods, respectively. The results showed that the essential oil containing neral geranial, geranyl acetate and α-pinene had good antioxidant potential (Ashrafi et al. 2017; Fallah et al. 2020). The essential oils from cultivated and wild *D. kotschyi* were tested for their inhibitory effects against 12 microbial strains by MIC and MBC tests. This activity was more marked against Gram-positive bacteria, while, essential oil from the wild was the most effective to halt *C. albicans* growth, the essential oil from crops was more marked against Gram-positive bacteria (*B. subtilis*) (Ghavam et al. 2021). Ashrafi et al. (2017) reported that *D. kotschyi* essential oil showed the greatest bactericidal activities against the highly susceptible strains of most Gram*-*positive organisms (except *E*. *faecalis*) with MIC values of 80–160 μg/mL and a few Gram-negative organisms; *Salmonella typhi*, *S. paratyphi* and *S. enterica* (80–160 μg/mL). In a comparative study, the antimicrobial activities of *D. polychaetum* Bornm., *D. kotschyi* and *D. multicaule* Montbret & Aucher ex Benth. were investigated wherein *D. kotschyi* essential oil with MIC of 200 μg/mL exhibited the strongest antimicrobial activity against *S. epidermidis* (Khodaei et al. 2018). *D. kotschyi* essential oil inhibitory effects against *K. pneumonia* as the third leading cause of hospital-acquired pneumonia were reported which can be replaced with conventional antibiotics such as amoxicillin (Shakib et al. 2018). In an investigation, the chemical composition and antibacterial efficacy of *D. kotschyi* essential oil were isolated by three different techniques (HD, solvent-free microwave extraction (SFME) and MAHD). The lowest MICs (2 mg/mL) were of the essential oil extracted by MAHD and SFME against *S. aureus*. However, the maximal limonene compounds were found in the essential oil obtained by HD (Moridi Farimani et al. 2017).

## *Hymenocrater* spp. (Lamiaceae)

*H. longiflorus* Benth. (Figure 1(K)) and *H. calycinus* (Boiss.) Benth. (Figure 1(L)) are termed Gol-e-Arvaneh in Persian (Morteza-Semnani et al. 2016). In Iranian traditional and folk medicine, it is optimally consumed for sedative, inflammation and skin antiallergenic (Asri et al. 2017). The antioxidant activities of essential oils besides polar and non-polar fractions of methanolic extract from *H. longiflorus* were estimated by DPPH and β-carotene-linoleic acid, respectively. According to DPPH assay results, polar extract exhibited better antioxidant activities due to lower EC_50_, while oxidation of linoleic acid was effectively inhibited by non-polar extracts (Ahmadi et al. 2010). *H. calycinus* essential oil was most effective to inhibit *S. aureus* (MIC = 0.8 mg/mL) growth. While it had no antifungal activity against any tested fungal strain (Morteza-Semnani et al. 2012). Ahmadi et al. (2010) showed that *S. aureus* (MIC = 40 μg/mL) was more sensitive to essential oil, and essential oil had significant inhibitory effects on *C. albicans* (MIC = 240 μg/mL) and *A. niger* (MIC = 480 μg/mL).

## *Mentha piperita* L. (Lamiaceae)

*M. piperita* (peppermint) (Figure 1(M)), a natural hybrid between spearmint (*M. spicata* L.) and water mint (*M. aquatica* L.) (Işcan et al. 2002), is traditionally used for migraine headache, antispasmodic, antiemetic, common cold symptoms, disinfectant and decongestant (Mikaili et al. 2012). Yazdani et al. (2019) demonstrated that *M. piperita* essential oil had a remarkable antioxidant effect comparable to BHT. According to β-carotene bleaching and DPPH radical tests, peppermint essential oil enriched with menthol and menthone displayed good antioxidant potential as compared with BHT and trolox (Fatemi et al. 2014). The *in vitro* antimicrobial property assessments demonstrated that *M. piperita* essential oil was more effective on Gram-positive (*S. epidermidis*, *B. subtilis* and *S. aureus*) than Gram-negative bacteria (*S. dysenteriae* and *K. pneumonia*) (Yazdani et al. 2019). Saharkhiz et al. (2012) reported that peppermint essential oil inhibited the biofilm formation of *C. albicans* and *C. dubliniensis* at concentrations up to 2 µL/mL using a 2,3-bis(2-methoxy-4-nitro-5-sulfo-phenyl)-2H-tetrazolium-5-carbox-anilide reduction assay.

## *Salvia mirzayanii* Rech.f. & Esfand. (Lamiaceae)

Salvia (*S. mirzayanii*) (Figure 1(N)) aerial parts have long been used to cure infections, inflammatory diseases, spasms, gastrointestinal disorders and diabetes (Sadat-Hosseini et al. 2017; Asadollahi et al. 2019). Oxygenated monoterpenes including β-thujone, 1,8-cineole and camphor (Izadi and Mirazi 2020) were the main constituents of Salvia essential oil displaying considerable antioxidant activities when compared to trolox (Omidpanah et al. 2015). Armana et al. (2012) investigated the chemical compositions and antimicrobial activity of essential oil from *S. mirzayanii* aerial plants against *B. subtilis*, *B. pumilus*, *E. faecalis*, *S. aureus*, *S. epidermidis*, *E. coli* and *A. niger.* Gram-positive bacteria (*B. subtilis* and *B. pumilus*) were the most sensitive microorganisms to Salvia essential oil (containing spathulenol, linalool and 1,8-cineole) with MIC = 1.87 mg/mL. Ghasemi et al. (2020) reported that chemical compounds and antimicrobial activities of *S. mirzayanii* essential oils were dependent on variety and environmental conditions. The essential oils of various plant species: *S. mirzayanii*, *Ocimum sanctum* L. (Lamiaceae), *A. sieberi* Besser., *Satureja khuzestanica* Jamzad (Lamiaceae), *Satureja bachtiarica* Bunge. (Lamiaceae) and *Z. multiflora* were tested for antimicrobial efficiency against oral bacteria; *Streptococcus mutans*, *C. albicans* and *E. faecalis* via broth micro-dilution method. The study revealed that *O. sanctum*, *A. sieberi* and *S. mirzayanii* essential oils rich in 1,8-cineole displayed strong antimicrobial effects (Zomorodian et al. 2015). Two years later, Zomorodian et al. (2017) investigated the antimicrobial effects of essential oil from *S. mirzayanii* leaves against some common pathogenic bacteria and fungi and found that Gram-negative (*E. faecalis*) bacteria were more sensitive than Gram-positive ones.

## *Satureja* spp. (Lamiaceae)

About 16 *Satureja* species are grown in Iran, of which, *S. bachtiarica* (Figure 1(O)), *S. khuzestanica* (Figure 1(P)) and *S. rechingeri* Jamzad (Figure 1(Q)) can be easily found in different parts of Iran (Jamzad 1996; Razzaghi-Abyaneh et al. 2013). In Iranian folk medicine, they treat cramps, muscle pains, nausea indigestion, diarrhoea and infectious diseases (Senatore et al. 1998; Bezić et al. 2009; Hadian et al. 2014). The aerial parts of these plants are used in traditional medicine as antibacterial, anti-inflammation, analgesic, antiseptic besides flavouring agents (Naghibi et al. 2005; Ghasemi Pirbalouti et al. 2010b). Alizadeh (2015) evaluated the antioxidant activity of essential oil and extract from *S. rechingeri* using DPPH and FRAP assays and revealed that antioxidant capacity of *S. rechingeri* extract was stronger than its essential oil due to high concentration of phenolic contents. Similarly, antioxidant activity of *S. rechingeri* essential oil solely and in combination with deuterium depleted water (DDW) has been evaluated using glutathione *S*-transferase (GST), lipid peroxidation (LP) and glutathione (GSH) methods respectively and based on data, essential oil solely or in combination with DDW showed a high antioxidant activity level as compared to BHT (Attaran et al. 2015; Fatemi et al. 2015; Rasooli et al. 2016). The therapeutic effects of DDW on various diseases in humans mainly cancer have been previously confirmed due to its great antioxidant potentials (Basov et al. 2019). Memarzadeh et al. (2020) characterized the impact of different extraction techniques vs. HD and microwave-assisted steam hydro-diffusion (MSHD) on phytochemical analysis and antioxidant capacity of *S. bachtiarica* essential oil. Although there was an equal amount of two principal components (carvacrol and thymol) present in the essential oils extracted by both methods, the antioxidant activity of essential oil extracted by MSHD was higher than that of HD. A comparative study assessed the chemical compositions and antioxidant activities of four essential oils (*S. khuzestanica*, *Oliveria decumbens* Vent. (Apiaceae) and *Thymus daenensis* Celak. (Lamiaceae) and their components). The results showed that antioxidant activity of *S. khuzestanica* essential oil was weaker than other tested essential oils (Saidi 2014). *S. khuzestanica* essential oil (IC_50_=28.71 μg/mL) exhibited the highest DPPH scavenging activity and strong antioxidant activity in β-carotene-linoleic acid assay (Saei-Dehkordi et al. 2012). Alizadeh (2015) assessed the antimicrobial effects of *S. rechingeri* oil against four microorganisms: *C. albicans* (ATCC 10231), *S. aureus* (ATCC 6538), *S. epidermidis* (ATCC 1435) and *E. coli* (ATCC 25922) by recording inhibition zones and MIC and revealed that *S. rechingeri* essential oil had a significant potential to inactivate the growth of all tested pathogens (MIC = 0.19 and 0.39 μL/mL) compared to standard antibiotics (tetracycline and amoxicillin). The *in vitro* antibacterial activities of *S. bachtiarica* essential oil were also evaluated against several bacterial species. The results indicated that *S. bachtiarica* essential oil containing carvacrol and thymol showed a stronger antimicrobial activity against *S. typhimurium* and *L. monocytogenes* (Ghasemi Pirbalouti et al. 2017). The inhibitory effect of *S. bachtiarica* essential oil against *P. aeruginosa* was also reported (Ghasemi Pirbalouti and Dadfar 2013). The antibacterial efficacy from *S. bachtiarica* and *T. daenensis* essential oils against *S. aureus* was considerably higher than that from *Dracocephalum multicaule* Montbr. & Auch. (Lamiaceae) and *Tanacetum polycephalum* Schultz-Bip (Asteraceae) *e*ssential oils (Ghasemi Pirbalouti et al. 2014). Previous researches demonstrated that *S. bachtiarica* essential oil had a therapeutic potentiality to treat *Helicobacter pylori* and *L. monocytogenes* (Falsafi et al. 2015; Fathi-moghaddam et al. 2020). The antibacterial activity of essential oils from *Satureja* species against some Gram-positive and negative bacteria was evaluated by disc diffusion method. The strongest antibacterial activities were observed in *S. khuzistanica* and *S. rechingeri* essential oils against *B. cereus* with MIC of 0.25 mg/mL. Interestingly, the ability of whole essential oils to prevent tested pathogens except *P. aeruginosa* was comparable to, or in most cases greater than, those of their pure main constituents (Hadian et al. 2012). Similarly, both *S. khuzistanica* and *S. rechingeri* essential oils possessed stronger antibacterial potential against Gram negative bacteria (*E. coli* and *S. flexneri*) than *S. bachtiarica* (Saharkhiz et al. 2016). *S. khuzestanica* essential oil showed strong antibacterial activity against *S. aureus* which was mainly correlated to presence of carvacrol (Rashidipour et al. 2016). Mahboubi and Kazempour (2016) evaluated *in vitro* antibacterial activity of *S. khuzestanica* essential oil, carvacrol and gentamicin and their synergistic effect on *E. coli.* They found that antibacterial activity of carvacrol was higher than essential oil. They also reported a considerable synergistic effect while using gentamicin with carvacrol and *S. khuzestanica* essential oil. Likewise, Hashemi and Khodaei (2020) conducted an *in vitro* study to assess the inhibitory potential of *S. khuzestanica* and *S. bachtiarica* essential oils solely or in combination against *Lactobacillus plantarum* LU5 growth as a probiotic culture or *S. flexneri*, and *E. coli.* Their results confirmed that the mixture of both essential oils had no synergistic effect on probiotics while it significantly prohibited the pathogen. The antibacterial effects of *S. khuzestanica* essential oil alone and in combination with both synthetic (ciprofloxacin fluconazole and amphotericin B) and natural (lysozyme) agents using fractional inhibitory concentration indices and MIC assays were studied against food-borne microorganisms. The results indicated that the interactive effects of combinations between essential oil and ciprofloxacin against *E. coli* and *S. typhimurium* were considerable (Saei-Dehkordi et al. 2012). Likewise, Golparvar et al. (2018) reported that essential oils of *S. hortensis* L. and *S. khuzestanica* showed antimicrobial activity against *S. aureus* and *C. albicans* (MIC = 0.1, 0.2 and 0.5 μL/mL, respectively).

## *Thymus* spp. (Lamiaceae)

*Thymus* contains 18 species in Iran, wherein *T. daenensis* (Figure 1(R)) and *T. kotschyanus* Boiss. & Hohen. (Figure 1(S)) are widely distributed in the central and southern parts of Iran (Jalas 1982; Mozafarian 1996). They treat common cold, high blood pressure, vomiting, heartburn, asthma and cough (Naghibi et al. 2005; Rahimmalek et al. 2009; Emami Bistgani and Sefidkon 2019). The antioxidant activities of *Thymus* species including *T. kotschyanus*, *T. daenensis* and *T. eriocalyx* Jalas have been addressed by various model systems; DPPH and β-carotene/linoleic acid. Their main phytochemical compositions were thymol, carvacrol and γ-terpinene. While the antioxidant effect of *T. daenensis* essential oil was higher than the other essential oils in DPPH assay and *T. eriocalyx* Jalas. exerted the greatest antioxidant activity in β-carotene/linoleic acid test (Amiri et al. 2012) and even greater than that of reference antioxidant (Alavi et al. 2010). In another comparative study, *T. vulgaris* L. exhibited the highest antioxidant activity as compared to other *Thymus* species (*T. daenensis* and *T. kotschyanus*) due to high thymol constituent (Mehran et al. 2016). However, the activity of *T. kotschyanus* leaves essential oil was lower than the positive control (BHT) (Shafaghat and Shafaghatlonba 2011). *T. kotschyanus* essential oil exerted notable antimicrobial effects on *C. albicans* and *B. cereus* (Ahmadi et al. 2015). Golkar et al. (2020) compared the antibacterial activities and chemical compositions of *Thymus* species and *Z. multiflora* through disk diffusion and broth microdilution assays against both Gram-positive and negative bacteria. They reported that *T. kotschyanus* and *Z. multiflora* had considerable antibacterial effects (MIC = 20 μg/mL) which might be attributed to high thymol and carvacrol levels. The antifungal activities and phytochemical compounds of *T. daenensis* essential oil were tested against two fungi. GC/MS analysis identified the major components: thymol, carvacrol and *p*-cymene, as well as the potent inhibitory effects at very broad spectrum against *A. fumigatus*, *A. niger* and *C. albicans* (Hadipanah and Khorami 2016; Mohammadi Gholami et al. 2018). Asbaghian et al. (2011) compared the antimicrobial activities of *Thymus* species (*T. caucasicus* Wild., *T. kotschyanus* and *T. vulgaris*) and showed that *T. vulgaris* with the highest thymol concentration (43.8%) had the highest antimicrobial activity on *E. coli* (MIC = 12.5 μg/mL), followed by *S. faecalis* (MIC = 25 μg/mL). According to Alamholo (2020), *T. daenensis* essential oil had the moderate antibacterial activity against *Streptococcus pyogenes*, *Micrococcus luteus* and *B. subtilis*, with MIC = 7.5 μg/mL and high-level activity against *E. faecalis* (MIC = 3.75 μg/mL), however, showed no activity against *S. typhi*, *P. aeruginosa*, *E. coli*, *Proteus mirabilis* and *S. boydii.*

## *Zataria multiflora* Boiss. (Lamiaceae)

*Z. multiflora* (Figure 1(T)), (Avishan-e-Shirazi), has been used for many decades as a flavouring ingredient in a number of Iranian cuisines (Gandomi et al. 2009; Basti et al. 2016). It has several traditional uses as an infusion, decoction or vapour to treat digestive problems, headache, common cold, migraine and bone pain (Safa et al. 2013; Nasab and Khosravi 2014). *Z. multiflora* essential oil containing the phenolic compounds particularly carvacrol and thymol demonstrated a high antioxidant activity through β-carotene-linoleic acid and DPPH assays (Fatemi et al. 2012; Dini et al. 2015), but lower than BHT (Hashemi et al. 2011). A number of different methods have been used to compare the radical-scavenging/antioxidant activity of essential oils isolated from *Z. multiflora* leaves and *Ferula assa-foetida* L. latex. The free radical scavenging activity estimated by inhibitory concentration ranged from 2.46 ± 0.75 to 4.58 ± 1.4 µg/mL for *Z. multiflora* which was more susceptible than that of *F. assa-foetida* essential oil (Kavoosi and Purfard 2013). The other related study results indicated that use of *Z. multiflora* essential oil could considerably modulate antioxidant/oxidative stress parameters including LP and GSH, as well as antioxidant enzymes such as GST (Dadkhah et al. 2014; Attaran et al. 2018). In a study by Sharififar et al. (2011), *Z. multiflora* essential oil exhibited strong scavenging activity, inhibiting LP at all tested doses (100, 200 and 400 µL/kg/day). Previously, several studies proved the antibacterial activities of *Z. multiflora* essential oil against different bacterial species. Fatemi et al. (2015) assessed the antibacterial potency of *Z. multiflora* essential oil (100 and 200 mg/kg b.w.) using disc diffusion, agar well diffusion, besides MIC and MBC determination assays as well as caecal ligation and puncture model. They reported that *S. aureus* and *B. cereus* (Gram-positive bacteria) were more sensitive to *Z. multiflora* essential oil than *E. coli* and *P. aeruginosa* (Gram-negative bacteria). Likewise, Rahimi et al. (2019) indicated that *Z. multiflora* essential oil inhibited *A. flavus* growth at 50–400 ppm concentrations. Even, Mahboubi et al. (2017) evaluated the antimicrobial activity of *Z. multiflora* essential oil and its main compounds; thymol, carvacrol and p-cymene against *P. aeruginosa.* The MICs results showed the equal growth inhibitory effects of both essential oil and its major compositions. The antimicrobial activity of *Z. multiflora* essential oil collected from Khorasan Province was analysed against seven microorganism strains. The lowest MICs were against *P. aeruginosa* (25 μg/mL) and *P. vulgaris* (25 μg/mL) and *S. cerevisiae*, *P. digitatum* and *A. niger* were the most resistant microorganisms (Avaei et al. 2015).

A comparative study on antibacterial activities of different essential oils against *Lactococcus garvieae*, as the causative agent of lactococcosis, indicated that the inhibitory effect of *Z. multiflora* essential oil (MIC = 7.8 μg/mL) were far stronger than *Rosmarinus officinalis* L. (Lamiaceae) (MIC = 15.6 μg/mL), *Anethum graveolens* L. (Apiaceae) (MIC = 62.4 μg/mL) and *Eucalyptus globulus* Labill. (Myrtaceae) (MIC = 250 μg/mL) (Mahmoodi et al. 2012). Similarly, the antibacterial property of *Z. multiflora* essential oil was superior to *Berberis vulgaris* L. (Berberidaceae) extract (Langroodi et al. 2018). Recently, Mahammadi Purfard and Kavoosi (2012) compared the effect of *Z. multiflora* essential oil and aqueous extract on inhibition of *P. aeruginosa*, *S. typhi*, *E. coli*, *K. pneumoniae*, *S. aureus*, *S. epidermidis*, *B. subtilis*, *A. niger* and *C. albicans* and demonstrated that *Z. multiflora* essential oil significantly inhibited the growth of all tested pathogens except *P. aeruginosa*, while, *Z. multiflora* extract was unable to inhibit the growth of all tested pathogens. Furthermore, the synergistic antibacterial activity of *Z. multiflora* essential oil in combination with monolaurin as a non-traditional antimicrobial agent was investigated. Consequently, the combination of these components revealed a more potent inhibitor against *L. monocytogenes* (Raeisi et al. 2016). In addition, the antibacterial property of *Z. multiflora* essential oil in combination with other antimicrobials has been well investigated. Rahnama et al. (2012) reported the enhanced synergistic antibacterial effect of *Z. multiflora* essential oil and nisin on *L. monocytogenes* through decrease in MIC and MBC values. Likewise, Javan (2016) indicated that the combination of *Z. multiflora* and *Trachyspermum ammi* L. (Apiaceae) essential oils exhibited a synergistic effect on the bacterial inhibition, and *B. cereus* was the most sensitive pathogen. For the first time, the application of silver nanoparticles as an antimicrobial agent in combination with *Z. multiflora* essential oil against a variety of pathogens *S. aureus*, methicillin-resistant *S. aureus* (MRSA), *S. epidermidis* and *P. aeruginosa* was investigated by Sheikholeslami et al. (2016). They confirmed that these compounds exerted additive effects against *S. epidermidis* and *S. aureus*. Moreover, Nasseri et al. (2016) demonstrated that *Z. multiflora* essential oil loaded with nanoliposomes showed higher antifungal effect on *Aspergillus* spp., *Rhizoctonia solani* and *Rhizopus stolonifer* than non-capsulated essential oil. Khatibi et al. (2017) also reported a significant increase in inhibitory effect of *Z. multiflora* essential oil against *E. coli* O157:H7 after encapsulation into nanoliposomes. Moreover, Shahabi et al. (2017) reported that although conversion of *Z. multiflora* essential oil to nanoemulsion could not significantly improve its antibacterial activity, but it enhanced its antibiofilm activity.

## *Zhumeria majdae* Rechinger f. & Wendelbo (Lamiaceae)

Mohrekhosh (*Z. majdae*) (Figure 1(U)) has been traditionally used as antiseptic, carminative and painkiller (Rechinger and Wendelbo 1967; Rechinger 1982; Safa et al. 2013). *Z. majdae* essential oils collected from different locations of Iran showed high antioxidant activity *in vitro* with DPPH (IC_50_=8.01) and β-carotene/linoleic assays (11.77 mg/mL). This study revealed a direct relationship between the geographical location and the antioxidant activity of *Z. majdae* essential oils (Saeidi et al. 2019). Mirzakhani et al. (2018) reported the inhibition effects of *Z. majdae* essential oil on some food-borne pathogenic bacteria; *B. cereus*, *E. faecalis* and *S. typhimurium.* They also signified that *Z. majdae* essential oil had inhibiting effects on all tested Gram-positive strains except *E. faecalis*.

## *Ziziphora clinopodioides* Lam. (Lamiaceae)

In Iranian folklore, different parts of kakuti-e kuhi (*Z. clinopodioides*) (Figure 1(V)), i.e., from leaves to roots were commonly used as spice and treatment of digestive system, cold and toothache (Asgharipour et al. 2016; Amiri et al. 2019). In the present study, antioxidant compounds (total phenol and flavonoid contents) and antioxidant activities of *Z. tenuior* L. and *Z. clinopodioides* essential oils were compared. The antioxidant analysis revealed that both essential oils considerably reduced the value of DPPH free radicals. The total phenolic compounds content in *Z. clinopodioides* essential oil (49 ± 1.4 mg quercetin/100 g oil) was higher than *Z. tenuior* essential oil (30.3 ± 0.1 mg gallic acid/100 g oil) (Hazrati et al. 2020). Shahbazi (2017) determined antioxidant activity of *Z. clinopodioides* essential oil was determined by four different tests; TBA and FRAP assays and revealed that the antioxidant effects of essential oils harvested from Kermanshah Province were highest. *Z. clinopodioides* essential oil exhibited a considerable antibacterial activity against food-borne pathogens, with MIC and MBC values ranging from 0.0012 to 0.0025 μL/mL, respectively, even much higher than records of tetracycline as a positive control, 2–2.5 μL/mL. Generally, Gram-negative bacteria are more resistant than Gram-positive ones (Shahbazi 2015). An *in vitro* study evaluated the antifungal properties of *C. cyminum*, *Z. clinopodioides* and *Nigella sativa* L. (Ranunculaceae) essential oils on *Aspergillus parasiticus*, which is able to produce aflatoxin as a toxic and carcinogenic metabolite. The findings from broth microdilution method, revealed that the tested fungi were most sensitive to *C. cyminum* (MIC_90_=1.6 mg/mL; MFC = 3.5 mg/mL) and *Z. clinopodioides* (MIC_90_=2.1 mg/mL; MFC = 5.5 mg/mL) (Khosravi et al. 2011). Furthermore, the comparative study on compositions and antibacterial activity of essential oils from leaf, flower and stem of *Z. clinopodioides* collected from four natural habitats in the western provinces of Iran was conducted. No significant difference was observed in antibacterial potential of essential oils isolated from different parts of the plant. The Gram-negative bacteria (*S. typhimurium* and *E. coli*) were more resistance to Gram-positive bacteria (*S. aureus*, *B. cereus*, *B. subtilis* and *L. monocytogenes*) in relation to essential oils. The main constituent of all essential oils except the essential oils collected from Kurdestan was carvacrol (Shahbazi 2017).

## *Rosa damascene* P. Mill. (Rosaceae)

Damask (*R. damascena*) (Figure 1(W)) is a hybrid between *R. gallica* L. and *R. phoenicia* Boiss. and was brought to European countries from Iran (Mahboubi 2016). This plant is widely used in varied industries; cosmetic, pharmaceutical and food for centuries (Georgiev and Stoyanova 2006). *R. damascena* essential oil exhibited a strong antioxidant activity as compared to BHT and trolox (Dadkhah et al. 2019; Kheirkhahan et al. 2020). Fatemi et al. (2020) revealed the positive treatment of animals with synergetic antioxidant effects of *R. damascena* essential oil and DDW due to regulation of oxidative stress/antioxidant parameters mainly; GSH, LP, GST and FRAP. Moreover, Afsari Sardari et al. (2019) showed that essential oil of fresh flowers had more antioxidant activity as compared to the spent flower essential oil. Damask essential oil exhibited antimicrobial activity against 20 microorganisms selected from both Gram-negative and positive bacteria. The essential oil exhibited the highest antimicrobial activity against *P. vulgaris* and *K. pneumonia* (Mahboubi et al. 2011). In a study, *R. damascena* essential oil with high alcoholic monoterpenes content; β-citronellol, geraniol, farnesol and geranyl acetate had the best antifungal and antibacterial effects (Moein et al. 2017). Kheirkhahan et al. (2020) studied antibacterial effect of *R. damascena* essential oil using the disc diffusion method against *S. aureus*, *E. coli* and *S. typhi* bacterial strains and reported that volatile oil obtained from Damask was effective against the three tested bacteria with MICs ranging from 500 to 1000 μL/mL.

## Conclusions and future perspectives

This review discussed the antioxidant and antimicrobial potencies of essential oils of some indigenous plant species from Iran commonly used in Iranian traditional medicine for a wide range of applications (Table 1). The 23 studied essential oils showed high antioxidant activity particularly, *C. carvi*, *C. cyminum*, *A. millefolium* and *T. daenensis* essential oils which exerted even greater effects than synthetic antioxidants; Trolox and BHT (Table 2). The antioxidant activity of these essential oils is related to their main chemical composition; primarily to the presence of polyphenolic compounds (carvacrol and thymol). These natural antioxidants could be effectually used as an adjuvant to shield our body against oxidative stress-related disorders including cardiovascular diseases, dementia, neurodegenerative diseases and cancer. However, it mandates a detailed study to explore their efficacy, safety and exact mechanism *in vivo* and in clinical trials.

Furthermore, this review revealed that essential oils isolated from the selected endemic medicinal plants possessed strong antibacterial activities against various bacterial and fungal pathogens. As depicted in Table 3, Gram-positive bacteria; *Staphylococcus* spp., *Bacillus* spp. and *Listeria monocytogenes* are more sensitive to *C. carvi*, *C. macropodum*, *C. cyminum*, *P. ferulacea*, *A. millefolium*, *Hymenocrater* spp., *Z. majdae* and *Z. clinopodioides* essential oils than Gram-negative bacteria such as *E. coli* and *S. enterica.* Likewise, the other essential oils have similar effects on inhibition of both Gram-negative and positive bacteria. Therefore, it can be safely concluded that antimicrobial activity of essential oils is highly dependent upon some parameters mainly essential oil type and microbial strains tested. Perhaps, the difference in antimicrobial potential of these plant species might stem from varying their phytochemical compounds. In addition, future studies should be focussed to determine antimicrobial activity mechanisms of these pure essential oils and their individual major compounds as well as activity enhancement in combination with other antimicrobial agents.

Unfortunately, the commercial use of these essential oils as antimicrobials is still a challenging scenario because of their poor solubility and stability. Moreover, the antimicrobial effects of essential oil can be reduced via exposure to light, heat and oxidation (Khatibi et al. 2017). Nevertheless, encapsulation of essential oils and their constituents seems to be an efficient solution to overcome such problems due to improvement in their oxidative-stability, thermo-stability, photo-stability, shelf-life and even biological activity as well as increasing their solubility (Stevanovic et al. 2020). Encapsulated *Z. multiflora* essential oil mentioned in this review is a good example. Thus, it is expected that this review would be helpful to adopt more efficient natural antimicrobial and antioxidant agents for pharmaceutical and food purposes.

## References

[CIT0001] Alavi L, Barzegar M, Jabari A, Naghdi BH. 2010. Effect of heat treatment on chemical composition and antioxidant property of *Thymus daenensis* essential oil. J Med Plants. 9(35):129–138.

[CIT0002] Amorati R, Foti MC, Valgimigli L. 2013. Antioxidant activity of essential oils. J Agric Food Chem. 61(46):10835–10847. 24156356 10.1021/jf403496k

[CIT0004] Afsari Sardari F, Mosleh G, Azadi A, Mohagheghzadeh A. 2019. Traditional and recent evidence on five phytopharmaceuticals from *Rosa damascena* Herrm. Pharmacogn Res. 6:77–84.

[CIT0005] Ahmadi F, Sadeghi S, Modarresi M, Abiri R, Mikaeli A. 2010. Chemical composition, *in vitro* anti-microbial, antifungal and antioxidant activities of the essential oil and methanolic extract of *Hymenocrater longiflorus* Benth., of Iran. Food Chem Toxicol. 48(5):1137–1144. 20132856 10.1016/j.fct.2010.01.028

[CIT0006] Ahmadi R, Alizadeh A, Ketabchi S. 2015. Antimicrobial activity of the essential oil of *Thymus kotschyanus* grown wild in Iran. Int J Biol Sci. 6:239–248.

[CIT0007] Ahmadi-Dastgerdi A, Ezzatpanah H, Asgary S, Dokhani S, Rahimi E. 2017. Phytochemical, antioxidant and antimicrobial activity of the essential oil from flowers and leaves of *Achillea millefolium* subsp. *millefolium*. J Essent Oil-Bear Plants. 20(2):395–409.

[CIT0008] Akbarzadeh T, Sabourian R, Saeedi M, Rezaeizadeh H, Khanavi M, Shams Ardekani MH. 2015. Liver tonics: review of plants used in Iranian traditional medicine. Asian Pac J Trop Biomed. 5(3):170–181.

[CIT0010] Alamholo M. 2020. Investigation of chemical composition, antibacterial and antioxidant activity of *Thymus daenensis* and *Thymus eriocalyx* essential oils against human pathogenic bacteria. J Med Microbiol. 8:148–154.

[CIT0011] Alfadda AA, Sallam RM. 2012. Reactive oxygen species in health and disease. J Biomed Biotechnol. 2012:936486. 22927725 10.1155/2012/936486 PMC3424049

[CIT0012] Alizadeh A. 2015. Essential oil composition, phenolic content, antioxidant, and antimicrobial activity of cultivated *Satureja rechingeri* Jamzad at different phenological stages. Z Naturforsch C J Biosci. 70(3–4):51–58. 25924231 10.1515/znc-2014-4121

[CIT0013] Amin GH. 1991. Iranian traditional medicinal plants. Tehran, Iran: Research Deputy of Health Ministry Publisher.

[CIT0014] Aminzare M, Amiri E, Abbasi Z, Hassanzad Azar H, Hashemi M. 2017. Evaluation of *in vitro* antioxidant characteristics of corn starch bioactive films incorporated with *Bunium persicum* and *Zataria multiflora* essential oils. Annu Res Rev Biol. 15(5):1–9.

[CIT0015] Amiri F, Gholipouri A, Kheirkhah M, Mirjalili MH. 2019. Study on ethnobotany and the effect of ecological factor on the yield of essential oil of *Ziziphora clinopodioides* Lam. (case study: Yazd Province). J Med Plants By Prod. 8:189–199.

[CIT0016] Amiri H. 2012. Essential oils composition and antioxidant properties of three *Thymus* species. Evid Based Complement Alternat Med. 2012:728065–728073. 21876714 10.1155/2012/728065 PMC3163135

[CIT0017] Amiri MS, Jabbarzadeh P, Akhondi M. 2012. An ethnobotanical survey of medicinal plants used by indigenous people in Zangelanlo district, Northeast Iran. J Med Plants Res. 6:749–753.

[CIT0018] Amiri MS, Joharchi MR. 2013. Ethnobotanical investigation of traditional medicinal plants commercialized in the markets of Mashhad, Iran. Avicenna J Phytomed. 3(3):254–271. 25050282 PMC4075713

[CIT0019] Amiri MS, Joharchi MR. 2016. Ethnobotanical knowledge of Apiaceae family in Iran: a review. Avicenna J Phytomed. 6(6):621–635. 28078243 PMC5206921

[CIT0020] Amirmohammadi M, Khajoenia S, Bahmani M, Rafieian-Kopaei M, Eftekhari Z, Qorbani M. 2014. *In vivo* evaluation of antiparasitic effects of *Artemisia abrotanum* and *Salvia officinalis* extracts on *Syphacia obvelata*, *Aspiculuris tetraptera* and *Hymenolepis nana* parasites. Asian Pac J Trop Dis. 4:S250–S254.

[CIT0021] Armana M, Azizi N, Yousefzadi M. 2012. Cytotoxicity, antimicrobial activity and composition of the essential oil from *Salvia mirzayanii* Rech. f. & Esfand from Iran. J Biol Active Prod Nat. 2:54–58.

[CIT0022] Arumugam G, Swamy MK, Sinniah UR. 2016. *Plectranthus amboinicus* (Lour.) Spreng: botanical, phytochemical, pharmacological and nutritional significance. Molecules. 21(4):369–395. 27043511 10.3390/molecules21040369 PMC6274163

[CIT0023] Asadollahi M, Firuzi O, Jamebozorgi FH, Alizadeh M, Jassbi AR. 2019. Ethnopharmacological studies, chemical composition, antibacterial and cytotoxic activities of essential oils of eleven *Salvia* in Iran. J Herb Med. 17:100250–100302.

[CIT0024] Asbaghian S, Shafaghat A, Zarea K, Kasimov F, Salimi F. 2011. Comparison of volatile constituents, and antioxidant and antibacterial activities of the essential oils of *Thymus caucasicus*, *T. kotschyanus* and *T. vulgaris*. Nat Prod Commun. 6:137–140. 21366065

[CIT0025] Asgharipour MR, Abjahan AA, Dahmarde M. 2016. Variability in *Ziziphora clinopodioides* subsp. *bungeana* (Juz.) based on morphological traits and essential oils profile. Not Bot Horti Agrobot. 44(1):189–194.

[CIT0026] Ashrafi B, Ramak P, Ezatpour B, Talei GR. 2017. Investigation on chemical composition, antimicrobial, antioxidant, and cytotoxic properties of essential oil from *Dracocephalum kotschyi* Boiss. Afr J Tradit Complement Altern Med. 14(3):209–217. 28480433 10.21010/ajtcam.v14i3.23 PMC5412227

[CIT0027] Asri Y, Sadeh-Hoseinabad Ghaini F, Vaziri A, Akbarzadeh M. 2017. Essential oil composition from *Hymenocrater calycinus* (Boiss.) Benth. in Iran. J Essent Oil-Bear Plants. 20(3):712–719.

[CIT0028] Attaran HR, Dini S, Fatemi F, Hesaraki S, Parhizkarie M, Dadkhah A. 2015. Hepatoprotective evaluation of Iranian *Satureja Rechingeri* essential oils against oxidative injuries induced by acetaminophen in Wistar rats. Int J Rev Life Sci. 5:204–210.

[CIT0029] Attaran HR, Fatemi F, Rasooli A, Dadkhah A, Mohammadi Malayeri MR, Dini S. 2018. *Zataria multiflora* essential oil prevent iron oxide nanoparticles-induced liver toxicity in rat model. J Med Plants By Prod. 7:15–24.

[CIT0030] Avaei A, Mohamadi Sani A, Mahmoodzadeh Vaziri B. 2015. Chemical composition and antimicrobial effect of the essential oil of *Zataria multiflora* Boiss. endemic in Khorasan-Iran. Asian Pac J Trop Dis. 5:181–185.

[CIT0031] Bagherifar S, Sourestani MM, Zolfaghari M, Mottaghipisheh J, Zomborszki ZP, Csupor D. 2019. Variation of chemical constituents and antiradical capacity of nine *Ferulago angulata* populations from Iran. Chem Biodivers. 16(10):e1900302. 31415713 10.1002/cbdv.201900302

[CIT0032] Bahadori MB, Dinparast L, Zengin G. 2016. The genus *Heracleum*: a comprehensive review on its phytochemistry, pharmacology, and ethnobotanical values as a useful herb. Comprehens Rev Food Sci Food Saf. 15(6):1018–1039. 10.1111/1541-4337.12222 33401836

[CIT0034] Bahmani M, Zargaran A, Rafieian-Kopaei M, Saki K. 2014. Ethnobotanical study of medicinal plants used in the management of diabetes mellitus in the Urmia, Northwest Iran. Asian Pac J Trop Dis. 7:S348–S354. 10.1016/S1995-7645(14)60257-1 25312149

[CIT0035] Bakkali F, Averbeck S, Averbeck D, Idaomar M. 2008. Biological effects of essential oils—a review. Food Chem Toxicol. 46(2):446–475. 17996351 10.1016/j.fct.2007.09.106

[CIT0036] Balouiri M, Sadiki M, Ibnsouda SK. 2016. Methods for *in vitro* evaluating antimicrobial activity: a review. J Pharm Anal. 6(2):71–79. 29403965 10.1016/j.jpha.2015.11.005 PMC5762448

[CIT0037] Basov A, Fedulova L, Baryshev M, Dzhimak S. 2019. Deuterium-depleted water influence on the isotope 2H/1H regulation in body and individual adaptation. Nutrients. 11(8):1903–1922. 10.3390/nu11081903 PMC6723318 31443167

[CIT0038] Basti AA, Gandomi H, Noori N, Khanjari A. 2016. Shirazi thyme (*Zataria multiflora* Boiss.) oils. in essential oils in food preservation. Flav Saf. 83:731–736.

[CIT0039] Bazdar M, Sadeghi H, Hosseini S. 2018. Evaluation of oil profiles, total phenols and phenolic compounds in *Prangos ferulacea* leaves and flowers and their effects on antioxidant activities. Biocatal Agric Biotechnol. 14:418–423.

[CIT0040] Benzie IFF, Strain JJ. 1996. The ferric reducing ability of plasma (FRAP) as a measure of “antioxidant power”: the FRAP assay. Anal Biochem. 239(1):70–76. 8660627 10.1006/abio.1996.0292

[CIT0041] Bertrand P, Cheong Sing A, Jacqueline Smadja A. 2005. Assessment of antioxidant activity of cane brown sugars by ABTS and DPPH radical scavenging assays: determination of their polyphenolic and volatile constituents. J Agric Food Chem. 53(26):10074–10079. 16366697 10.1021/jf0517703

[CIT0042] Bezić N, Šamanić I, Dunkić V, Besendorfer V, Puizina J. 2009. Essential oil composition and internal transcribed spacer (its) sequence variability of four south-Croatian *Satureja* species (Lamiaceae). Molecules. 14(3):925–938. 19255551 10.3390/molecules14030925 PMC6253779

[CIT0043] Bondet V, Brand-Williams W, Berset CL. 1997. Kinetics and mechanisms of antioxidant activity using the DPPH free radical method. LWT Food Sci Technol. 30(6):609–615.

[CIT0044] Borges A, Ferreira C, Saavedra MJ, Simões M. 2013. Antibacterial activity and mode of action of ferulic and gallic acids against pathogenic bacteria. Microb Drug Resist. 19(4):256–265. 23480526 10.1089/mdr.2012.0244

[CIT0045] Borges A, Simões LC, Saavedra MJ, Simões M. 2014. The action of selected isothiocyanates on bacterial biofilm prevention and control. Int Biodeterior Biodegrad. 86:25–33.

[CIT0046] Buso P, Manfredini S, Reza Ahmadi-Ashtiani H, Sciabica S, Buzzi R, Vertuani S, Baldisserotto A. 2020. Iranian medicinal plants: from ethnomedicine to actual studies. Medicina. 56(3):97–150. 10.3390/medicina56030097 PMC7143749 32110920

[CIT0047] Caleja C, Barros L, Antonio AL, Oliveira MB, Ferreira IC. 2017. A comparative study between natural and synthetic antioxidants: evaluation of their performance after incorporation into biscuits. Food Chem. 216:342–346. 27596429 10.1016/j.foodchem.2016.08.075

[CIT0048] Chouhan S, Sharma K, Guleria S. 2017. Antimicrobial activity of some essential oils—present status and future perspectives. Medicines. 4(3):58–79. 10.3390/medicines4030058 PMC5622393 28930272

[CIT0049] Chrysargyris A, Mikallou M, Petropoulos S, Tzortzakis N. 2020. Profiling of essential oils components and polyphenols for their antioxidant activity of medicinal and aromatic plants grown in different environmental conditions. Agron. 10(5):727–755.

[CIT0050] Cox SD, Mann CM, Markham JL, Bell HC, Gustafson JE, Warmington JR, Wyllie SG. 2000. The mode of antimicrobial action of the essential oil of *Melaleuca alternifolia* (tea tree oil). J Appl Microbiol. 88(1):170–175. 10735256 10.1046/j.1365-2672.2000.00943.x

[CIT0051] Dadkhah A, Allameh A, Khalafi H, Ashrafihelan J. 2011. Inhibitory effects of dietary caraway essential oils on 1,2-dimethylhydrazine-induced colon carcinogenesis is mediated by liver xenobiotic metabolizing enzymes. Nutr Cancer. 63(1):46–54. 21108126 10.1080/01635581.2010.516473

[CIT0052] Dadkhah A, Fatemi F, Malayeri MRM, Ashtiyani MHK, Noureini SK, Rasooli A. 2019. Considering the effect of *Rosa damascena* Mill. essential oil on oxidative stress and Cox-2 gene expression in the liver of septic rats. Turk J Pharm Sci. 16(4):416–425. 32454744 10.4274/tjps.galenos.2018.58815 PMC7227890

[CIT0053] Dadkhah A, Fatemi F, Mohammadi Malayeri MR, Rasooli A. 2014. Cancer chemopreventive effect of dietary *Zataria multiflora* essential oils. Turk J Biol. 38:930–939.

[CIT0054] Dadkhah A, Fatemi F, Mohammadi Malayeri MR, Torabi F, Sarbazi M, Dini S. 2018. Potential protective effect of pretreatment with caraway essential oil *in vivo* model of iron nanoparticle-induced liver injury. J Med Plants By Prod. 7:145–152.

[CIT0056] Dehghan H, Sarrafi Y, Salehi P. 2016. Antioxidant and antidiabetic activities of herbal plants from Hyrcania region. Iran J Food Drug Anal. 24(1):179–188. 28911402 10.1016/j.jfda.2015.06.010 PMC9345419

[CIT0058] Dhifi W, Bellili S, Jazi S, Bahloul N, Mnif W. 2016. Essential oils' chemical characterization and investigation of some biological activities: a critical review. Medicines. 3(4):25–41. 10.3390/medicines3040025 PMC5456241 28930135

[CIT0059] Dini S, Dadkhah A, Fatemi F. 2015. Biological properties of Iranian *Zataria multiflora* essential oils: a comparative approach. Electron J Biol. 11:57–62.

[CIT0060] Dolatkhahi M, Dolatkhahi A, Nejad JB. 2014. Ethnobotanical study of medicinal plants used in Arjan–Parishan protected area in Fars Province of Iran. Avicenna J Phytomed. 4(6):402–412. 25386404 PMC4224954

[CIT0061] Ebrahimabadi AH, Djafari-Bidgoli Z, Mazoochi A, Kashi FJ, Batooli H. 2010. Essential oils composition, antioxidant and antimicrobial activity of the leaves and flowers of *Chaerophyllum macropodum* Boiss. Food Control. 21(8):1173–1178.

[CIT0062] Ehsani A, Rezaeiyan A, Hashemi M, Aminzare M, Jannat B, Afshari A. 2019. Antibacterial activity and sensory properties of *Heracleum persicum* essential oil, nisin, and *Lactobacillus acidophilus* against *Listeria monocytogenes* in cheese. Vet World. 12(1):90–96. 30936660 10.14202/vetworld.2019.90-96 PMC6431807

[CIT0063] Ekhtelat M, Bahrani Z, Siahpoosh A, Ameri A. 2019. Evaluation of antibacterial effects of *Mentha spicata* L., *Cuminum cyminum* L. and *Mentha longifolia* L. essential oils individually and in combination with sodium benzoate against *Escherichia coli* O157: H7 and *Listeria monocytogenes*. Jundishapur J Nat Pharm Prod. 14(3):e59092–e59100.

[CIT0064] Emami Bistgani ZE, Sefidkon F. 2019. Review on ethnobotany, phytochemical, molecular and pharmacological activity of *Thymus daenensis* Celak. Biocatal Agric Biotechnol. 22:101400–101430.

[CIT0065] Fallah S, Mouguee S, Rostaei M, Adavi Z, Lorigooini Z. 2020. Chemical compositions and antioxidant activity of essential oil of wild and cultivated *Dracocephalum kotschyi* grown in different ecosystems: a comparative study. Ind Crops Prod. 143:111885–111894.

[CIT0066] Falsafi T, Moradi P, Mahboubi M, Rahimi E, Momtaz H, Hamedi B. 2015. Chemical composition and anti-*Helicobacter pylori* effect of *Satureja bachtiarica* Bunge essential oil. Phytomedicine. 22(1):173–177. 25636887 10.1016/j.phymed.2014.11.012

[CIT0067] Fatemi F, Allameh A, Khalafi H, Rajaee R, Davoodian N, Rezaei M. 2011. Biochemical properties of γ-irradiated caraway essential oils. J Food Biochem. 35(2):650–662.

[CIT0068] Fatemi F, Allameh A, Khalafi H, Rezaei MB, Seyhoon M. 2010. The effect of essential oils and hydroalcoholic extract of caraway seed on oxidative stress parameters in rats suffering from acute lung inflammation before and after γ-irradiation. J Med Aromat. 25:441–455.

[CIT0069] Fatemi F, Asri Y, Rasooli I, Alipoor SD, Shaterloo M. 2012. Chemical composition and antioxidant properties of γ-irradiated Iranian *Zataria multiflora* extracts. Pharm Biol. 50(2):232–238. 22092051 10.3109/13880209.2011.596208

[CIT0070] Fatemi F, Dadkhah A, Akbarzadeh K, Dini S, Hatami S, Rasooli A. 2015. Hepatoprotective effects of deuterium depleted water (DDW) adjuvant with *Satureja rechingeri* essential oils. Electron J Biol. 11:23–32.

[CIT0071] Fatemi F, Dadkhah A, Rezaei M, Dini S. 2013. Effect of γ-irradiation on the chemical composition and antioxidant properties of cumin extracts. Food Biochem. 37(4):432–439.

[CIT0072] Fatemi F, Dini S, Ddkhah A, Reza ZM. 2015. Considering the antibacterial activity of *Zataria multiflora* Boiss. essential oil treated with γ-irradiation *in vitro* and *in vivo* systems. Radiat Phys Chem. 106:145–150.

[CIT0073] Fatemi F, Dini S, Rezaei MB, Dadkhah A, Dabbagh R, Naij S. 2014. The effect of γ-irradiation on the chemical composition and antioxidant activities of peppermint essential oil and extract. J Essent Oil Res. 26(2):97–104.

[CIT0074] Fatemi F, Golbodagh A, Hojihosseini R, Dadkhah A, Akbarzadeh K, Dini S, Malayeri MR. 2020. Anti-inflammatory effects of deuterium-depleted water plus *Rosa damascena* Mill. essential oil via cyclooxygenase-2 pathway in rats. Turk J Pharm Sci. 17(1):99–107. 32454767 10.4274/tjps.galenos.2018.24381 PMC7227869

[CIT0217] Fathi Moghaddam E, Shakerian A, Sharafati Chaleshtori R, Rahimi E. 2020. Chemical composition and antioxidant properties and antimicrobial effects of *Satureja bachtiarica* Bunge. and *Echinophora platyloba* DC. essential oils against Listeria monocytogenes. J Medicinal Plants By- Products. 9:47–58.

[CIT0075] Firuzi O, Asadollahi M, Gholami M, Javidnia K. 2010. Composition and biological activities of essential oils from four *Heracleum* species. Food Chem. 122(1):117–122.

[CIT0076] Gachkar L, Yadegari D, Rezaei MB, Taghizadeh M, Astaneh SA, Rasooli I. 2007. Chemical and biological characteristics of *Cuminum cyminum* and *Rosmarinus officinalis* essential oils. Food Chem. 102(3):898–904.

[CIT0077] Gandomi H, Misaghi A, Basti AA, Bokaei S, Khosravi A, Abbasifar A, Javan AJ. 2009. Effect of *Zataria multiflora* Boiss. essential oil on growth and aflatoxin formation by *Aspergillus flavus* in culture media and cheese. Food Chem Toxicol. 47(10):2397–2400. 19477213 10.1016/j.fct.2009.05.024

[CIT0078] Gavanji S, Mohammadi E, Larki B, Bakhtari A. 2014. Antimicrobial and cytotoxic evaluation of some herbal essential oils in comparison with common antibiotics in bioassay condition. Integr Med Res. 3(3):142–152. 28664090 10.1016/j.imr.2014.07.001 PMC5481736

[CIT0079] Georgiev E, Stoyanova A. 2006. Handbook of the specialists in aroma industry. Plovdiv: Bulgarian National Association of Essential Oils, Perfumery and Cosmetics.

[CIT0080] Ghasemi PA, Momeni M, Bahmani M. 2013. Ethnobotanical study of medicinal plants used by Kurd tribe in Dehloran and Abdanan districts, Ilam Province, Iran. Afr J Tradit Complement Altern Med. 10(2):368–385. 24146463 10.4314/ajtcam.v10i2.24 PMC3746586

[CIT0218] Ghasemi G, Alirezalu A, Ghosta Y, Jarrahi A, Safavi SA, Abbas-Mohammadi M, Barba FJ, Munekata PE, Domínguez R, Lorenzo JM. 2020. Composition, antifungal, phytotoxic, and insecticidal activities of *Thymus kotschyanus* essential oil. Molecules. 25(5):1152–1170. 10.3390/molecules25051152 PMC7179150 32143475

[CIT0081] Ghasemi Pirbalouti A, Izadi A, Malek Poor F, Hamedi B. 2016. Chemical composition, antioxidant and antibacterial activities of essential oils from *Ferulago angulata*. Pharm Biol. 54(11):2515–2520. 27102982 10.3109/13880209.2016.1162816

[CIT0082] Ghasemi Pirbalouti A, Nourafcan H, Solyamani-Babadi E. 2017. Variation in chemical composition and antibacterial activity of essential oils from Bakhtiari savory (*Satureja bachtiarica* Bunge.). J Essent Oil-Bear Plants. 20(2):474–484.

[CIT0083] Ghasemi Pirbalouti AG, Dadfar S. 2013. Chemical constituents and antibacterial activity of essential oil of *Satureja bachtiarica* (Lamiaceae). Acta Pol Pharm Drug Res. 70:933–938. 24147374

[CIT0084] Ghasemi Pirbalouti AG, Koohpayeh A, Karimi I. 2010a. The wound healing activity of flower extracts of *Punica granatum* and *Achillea kellalensis* in Wistar rats. Acta Pol Pharm. 67(1):107–110. 20210088

[CIT0085] Ghasemi Pirbalouti AG, Malekpoor F, Enteshari S, Yousefi M, Momtaz H, Hamedi B. 2010b. Antibacterial activity of some folklore medicinal plants used by Bakhtiari tribal in Southwest Iran. Int J Biol. 2:55–63.

[CIT0086] Ghasemi Pirbalouti AG, Neshat SH, Rahimi E, Hamedi B, Malekpoor F. 2014. Chemical composition and antibacterial activity of essential oils of Iranian herbs against *Staphylococcus aureus* isolated from milk. Int J Food Prop. 17(9):2063–2071.

[CIT0087] Ghavam M, Manconi M, Manca ML, Bacchetta G. 2021. Extraction of essential oil from *Dracocephalum kotschyi* Boiss. (Lamiaceae), identification of two active compounds and evaluation of the antimicrobial properties. J Ethnopharmacol. 267:113513–113541. 33172599 10.1016/j.jep.2020.113513

[CIT0088] Gilca M, Stoian I, Atanasiu V, Virgolici B. 2007. The oxidative hypothesis of senescence. J Postgrad Med. 53(3):207–213. 17700000 10.4103/0022-3859.33869

[CIT0089] Golkar P, Mosavat N, Jalali SA. 2020. Essential oils, chemical constituents, antioxidant, antibacterial and *in vitro* cytotoxic activity of different *Thymus* species and *Zataria multiflora* collected from Iran. S Afr J Bot. 130:250–258.

[CIT0090] Golparvar AR, Gheisari MM, Hadipanah A, Khorrami M. 2018. Antibacterial, antifungal properties and chemical composition of essential oils of *Satureja hortensis* L. and *Satureja khuzestanica* Jamzad. J Med Herb. 8:243–249.

[CIT0091] Hadian J, Akramian M, Heydari H, Mumivand H, Asghari B. 2012. Composition and *in vitro* antibacterial activity of essential oils from four *Satureja* species growing in Iran. Nat Prod Res. 26(2):98–108. 21827283 10.1080/14786419.2010.534734

[CIT0092] Hadian J, Esmaeili H, Nadjafi F, Khadivi-Khub A. 2014. Essential oil characterization of *Satureja rechingeri* in Iran. Ind Crops Prod. 61:403–409.

[CIT0093] Hadipanah A, Khorami M. 2016. Antimicrobial activity and chemical composition of *Thymus vulgaris* and *Thymus daenensis* essential oils. Adv Pharm J. 4:101–107.

[CIT0094] Haghi G, Hatami A, Ghasian F, Hoseini H. 2010. Antioxidant activity evaluation and essential oil analysis of *Chaerophyllum macropodum* Boiss. from central Iran. J Essent Oil-Bear Plants. 13(4):489–495.

[CIT0095] Haidari F, Seyed-Sadjadi N, Taha-Jalali M, Mohammed-Shahi M. 2011. The effect of oral administration of *Carum carvi* on weight, serum glucose, and lipid profile in streptozotocin-induced diabetic rats. Saudi Med J. 32(7):695–700. 21748206

[CIT0096] Hariri A, Ouis N, Bouhadi D, Benatouche Z. 2018. Characterization of the quality of the steamed yoghurts enriched by dates flesh and date powder variety H'loua. Banat's J Biotechnol. IX(17):31–39.

[CIT0219] Hashemi MB, Niakousari M, Saharkhiz MJ, Eskandari MH. 2011. Influence of *Zataria multiflora* Boiss. essential oil on oxidative stability of sunflower oil. Eur J Lipid Sci Technol. 113(12):1520–1526.

[CIT0097] Hashemi SM, Khodaei D. 2020. Antimicrobial activity of *Satureja khuzestanica* Jamzad and *Satureja bachtiarica* Bunge. essential oils against *Shigella flexneri* and *Escherichia coli* in table cream containing *Lactobacillus plantarum* LU5. Food Sci Nutr. 8(11):5907–5915. 33282242 10.1002/fsn3.1871 PMC7684589

[CIT0220] Hazrati S, Ebadi MT, Mollaei S, Khurizadeh S. 2019. Evaluation of volatile and phenolic compounds, and antioxidant activity of different parts of *Ferulago angulata* (schlecht.) Boiss. Ind Crops Prod. 140(111589):111589.

[CIT0098] Hazrati S, Govahi M, Sedaghat M, Kashkooli AB. 2020. A comparative study of essential oil profile, antibacterial and antioxidant activities of two cultivated *Ziziphora* species (*Z. clinopodioides* and *Z. tenuior*). Ind Crops Prod. 157:112942–112949.

[CIT0099] Hemati A, Azarnia M, Angaji AH. 2010. Medicinal effects of *Heracleum persicum* (Golpar). Middle-East J Sci Res. 5:174–176.

[CIT0100] Heydari P, Yavari M, Adibi P, Asghari G, Ghanadian SM, Dida GO, Khamesipour F. 2019. Medicinal properties and active constituents of *Dracocephalum kotschyi* and its significance in Iran: a systematic review. Evid Based Complement Alternat Med. 2019:1–14. 10.1155/2019/9465309 PMC6526565 31198431

[CIT0101] Hyldgaard M, Mygind T, Meyer RL. 2012. Essential oils in food preservation: mode of action, synergies, and interactions with food matrix components. Front Microbiol. 3:12–37. 22291693 10.3389/fmicb.2012.00012 PMC3265747

[CIT0102] Ighodaro OM, Akinloye OA. 2018. First line defence antioxidants—superoxide dismutase (SOD), catalase (CAT) and glutathione peroxidase (GPX): their fundamental role in the entire antioxidant defence grid. Alexandria J Med. 54(4):287–293.

[CIT0103] Iqbal J, Abbasi BA, Mahmood T, Kanwal S, Ali B, Shah SA, Khalil AT. 2017. Plant-derived anticancer agents: a green anticancer approach. Asian Pac J Trop Biomed. 7(12):1129–1150.

[CIT0104] Işcan G, Kirimer N, Kürkcüoğlu M, Başer KHC, Demirci F. 2002. Antimicrobial screening of *Mentha piperita* essential oils. J Agric Food Chem. 50(14):3943–3946. 12083863 10.1021/jf011476k

[CIT0105] Izadi Z, Mirazi N. 2020. Identification of chemical compounds and evaluation of antioxidant and antimicrobial properties of sage (*Salvia officinalis* L.) essential oil at different harvest times. Qom Univ Med Sci J. 14(9):1–5.

[CIT0106] Jahantab E, Hatami E, Sayadian M, Salahi Ardakani A. 2018. Ethnobotanical study of medicinal plants of Boyer Ahmad and Dena regions in Kohgiluyeh and Boyer Ahmad province. Iran Adv Herb. 3:12–22.

[CIT0107] Jalas J. 1982. Flora Iranica 1982, No. 150. New York: Springer; p. 536–538.

[CIT0108] Jamzad Z. 1996. *Satureja rechingeri* (Labiatae)—a new species from Iran. Ann Nat Mus Wien B Für Bot Zool. 1:75–77.

[CIT0109] Javan AJ. 2016. Combinational effects of *Trachyspermum ammi* and *Zataria multiflora* Boiss essential oils on some pathogenic food-borne bacteria. Koomesh. 17:374–383.

[CIT0110] Johri RK. 2011. *Cuminum cyminum* and *Carum carvi*: an update. Pharmacogn Rev. 5(9):63–72. 22096320 10.4103/0973-7847.79101 PMC3210012

[CIT0111] Karim MH, Karbasi A, Mohamadzadeh SH. 2020. Marketing strategies and export of Iranian medicinal plants. J Med Plants By Prod. 9:101–111.

[CIT0112] Kavoosi G, Purfard AM. 2013. Scolicidal effectiveness of essential oil from *Zataria multiflora* and *Ferula assa-foetida*: disparity between phenolic monoterpenes and disulphide compounds. Comp Clin Pathol. 22(5):999–1005.

[CIT0113] Kazemi M, Dakhili M, Dadkhah A, Yasrebifar Z, Larijani K. 2011. Composition, antimicrobial and antioxidant activities of the essential oil of *Artemisia kermanensis* Podl., an endemic species from Iran. J Med Plants Res. 5:4481–4486.

[CIT0114] Kazemi M. 2015. Chemical composition and antimicrobial, antioxidant activities and anti-inflammatory potential of *Achillea millefolium* L., *Anethum graveolens* L., and *Carum copticum* L. essential oils. J Herb Med. 5(4):217–222.

[CIT0115] Keshavarz A, Minaiyan M, Ghannadi A, Mahzouni P. 2012. Effects of *Carum carvi* L. (caraway) extract and essential oil on TNBS-induced colitis in rats. Res Pharm Sci. 8:1–8. PMC3895295 24459470

[CIT0116] Khajehie N, Golmakani MT, Eblaghi M, Eskandari MH. 2017. Evaluating the effects of microwave-assisted hydrodistillation on antifungal and radical scavenging activities of *Oliveria decumbens* and *Chaerophyllum macropodum* essential oils. J Food Prot. 80(5):783–791. 10.4315/0362-028X.JFP-16-428 28371590

[CIT0117] Khatibi SA, Misaghi A, Moosavy MH, Basti AA, Koohi MK, Khosravi P, Haghirosadat F. 2017. Encapsulation of *Zataria multiflora* Bioss. essential oil into nanoliposomes and *in vitro* antibacterial activity against *Escherichia coli* o157: H7. J Food Process Preserv. 41(3):e12955–e12965.

[CIT0118] Kheirkhahan P, Ghavami M, Sharifan A. 2020. Chemical composition, antioxidant activity and antimicrobial effect of *Rosa damascena* Mill. essential oil against *Staphylococcus aureus*, *Escherichia coli* and *Salmonella typhi*. J Food Biosci Technol. 10:63–74.

[CIT0119] Khodaei M, Amanzadeh Y, Faramarzi MA, Hamedani MP. 2018. Chemical analysis and anti-bacterial effect of essential oils from three different species of *Dracocephalum* in Iran. Am J Essent Oil. 6:31–34.

[CIT0120] Khosravi AR, Shokri H, Minooeianhaghighi M. 2011. Inhibition of aflatoxin production and growth of *Aspergillus parasiticus* by *Cuminum cyminum*, *Ziziphora clinopodioides*, and *Nigella sativa* essential oils. Foodborne Pathog Dis. 8(12):1275–1280. 21861703 10.1089/fpd.2011.0929

[CIT0121] Kousha A, Bayat M. 2012. Bactericidal and fungicidal activity of methanolic extracts of *Heracleum persicum* Desf. ex Fischer against some aquatic and terrestrial animal pathogens. Int J Pharmacol. 8(7):652–656.

[CIT0122] Kumar Y, Prakash O, Tripathi H, Tandon S, Gupta MM, Rahman LU, Lal RK, Semwal M, Darokar MP, Khan F. 2018. Aroma Db: a database of medicinal and aromatic plant’s aroma molecules with phytochemistry and therapeutic potentials. Front Plant Sci. 13:1081–1092. 10.3389/fpls.2018.01081 PMC6099104 30150996

[CIT0123] Ladan Moghadam AR. 2016. Chemical composition and antioxidant activity *Cuminum cyminum* L. essential oils. Int J Food Prop. 19(2):438–442.

[CIT0124] Langroodi AM, Fathabad AE, Moulodi F, Mashak Z, Abad MA. 2018. Antioxidant and antimicrobial activities of aqueous and ethanolic extracts of barberry and *Zataria multiflora* Boiss. essential oil against some food-borne bacteria. J Kermanshah Univ Med Sci. 22(2):e83087–e83094.

[CIT0125] Li R, Zhenquan J, Trush MA. 2016. Defining ROS in biology and medicine. React Oxyg Species (Apex). 1(1):9–21. 29707643 10.20455/ros.2016.803 PMC5921829

[CIT0126] Mahammadi Purfard AM, Kavoosi G. 2012. Chemical composition, radical scavenging, antibacterial and antifungal activities of *Zataria multiflora* Bioss. essential oil and aqueous extract. J Food Hyg Saf. 32:326–332.

[CIT0127] Mahboubi M, Heidarytabar R, Mahdizadeh E. 2017. Antibacterial activity of *Zataria multiflora* essential oil and its main components against *Pseudomonas aeruginosa*. Herba Polonica. 63(3):18–24.

[CIT0128] Mahboubi M, Kazempour N, Khamechian T, Fallah MH, Kermani MM. 2011. Chemical composition and antimicrobial activity of *Rosa damascena* Mill. essential oil. J Biol Active Prod Nat. 1(1):19–26.

[CIT0129] Mahboubi M, Kazempour N. 2016. The antibacterial activity of *Satureja khuzestanica* essential oil against clinical isolates of *E. coli*. Jundishapur J Nat Pharm Prod. 11(2):e30034–e30040.

[CIT0130] Mahboubi M. 2016. *Rosa damascena* as holy ancient herb with novel applications. J Tradit Complement Med. 6(1):10–16. 26870673 10.1016/j.jtcme.2015.09.005 PMC4737971

[CIT0131] Mahmoodi A, Roomiani L, Soltani M, Basti AA, Kamali A, Taheri S. 2012. Chemical composition and antibacterial activity of essential oils and extracts from *Rosmarinus officinalis*, *Zataria multiflora*, *Anethum graveolens* and *Eucalyptus globulus*. Glob Veter. 9:73–79.

[CIT0132] Mahomoodally MF, Chintamunnee V. 2012. Herbal medicine commonly used against infectious diseases in the tropical island of Mauritius. J Herb Med. 2(4):113–125.

[CIT0133] Majidi Z, Bina F, Kahkeshani N, Rahimi R. 2020. *Bunium persicum*: a review of ethnopharmacology, phytochemistry, and biological activities. Eur J Integr Med. 5:150–176.

[CIT0134] Mazandarani M, Osia N, Mosavi AK, Bayat H. 2013. Ecological requirements, antioxidant activity and new chemotype essential oil from *Achillea millefolium* L. and *Achillea micrantha* Wild. in North of Iran (Golestan Province). J Med Plants By Prod. 2:33–42.

[CIT0135] Mazidi S, Rezaei K, Golmakani MT, Sharifan A, Rezazadeh S. 2012. Antioxidant activity of essential oil from Black Zira (*Bunium persicum* Boiss.) obtained by microwave-assisted hydro distillation. J Agric Sci Technol. 14:1013–1022.

[CIT0136] Mehran M, Hoseini H, Hatami A, Taghizade M. 2016. Investigation of components of seven species of thyme essential oils and comparison of their antioxidant properties. J Med Plants. 2:134–140.

[CIT0137] Memarzadeh SM, Gholami A, Pirbalouti AG, Masoum S. 2020. Bakhtiari savory (*Satureja bachtiarica* Bunge.) essential oil and its chemical profile, antioxidant activities, and leaf micromorphology under green and conventional extraction techniques. Ind Crops Prod. 154:112719–112730.

[CIT0138] Mikaili P, Shayegh J, Sarahroodi S, Sharifi M. 2012. Pharmacological properties of herbal oil extracts used in Iranian traditional medicine. Adv Environ Biol. 6:153–158.

[CIT0139] Miller HE. 1971. A simplified method for the evaluation of antioxidants. J Am Oil Chem Soc. 48(2):91–92.

[CIT0140] Mirdeilami SZ, Barani H, Mazandarani M, Heshmati GA. 2011. Ethnopharmacological survey of medicinal plants in Maraveh Tappeh region, north of Iran. Iran J Plant Physiol. 2:325–336.

[CIT0141] Mirzakhani M, Ekrami M, Moini S. 2018. Chemical composition, total phenolic content and antimicrobial activities of *Zhumeria majdae*. J Food Bioprocess Eng. 1:47–52.

[CIT0142] Moazzami Farida SH, Ajani Y, Sadr M, Mozaffarian V. 2018. Ethnobotanical applications and their correspondence with phylogeny in Apiaceae–Apioideae. Res J Pharmacogn. 5:79–97.

[CIT0143] Moein M, Zomorodian K, Almasi M, Pakshir K, Zarshenas MM. 2017. Preparation and analysis of *Rosa damascena* essential oil composition and antimicrobial activity assessment of related fractions. Iran J Sci Technol Trans Sci. 41(1):87–94.

[CIT0144] Moghaddam M, Mehdizadeh L, Mirzaei Najafgholi H, Ghasemi Pirbalouti A. 2018. Chemical composition, antibacterial and antifungal activities of seed essential oil of *Ferulago angulata*. Int J Food Prop. 21(1):158–170.

[CIT0146] Mohammadi Gholami AN, Shiravand S, Ebrahimi K. 2018. Chemical composition, anti-fungal properties, anti-oxidant activity and cytotoxicity of *Thymus daenensis* Celak. essential oil. Eco-Phytochem J Med Plants. 6:56–68.

[CIT0222] Mojaddar Langroodi A, Tajik H, Mehdizadeh T. 2019. Antibacterial and antioxidant characteristics of *Zataria multiflora* Boiss. essential oil and hydroalcoholic extract of *Rhus coriaria* L. J Food Qual Hazards Control. 6(1):16–24.

[CIT0147] Moridi Farimani M, Mirzania F, Sonboli A, Moghaddam FM. 2017. Chemical composition and antibacterial activity of *Dracocephalum kotschyi* essential oil obtained by microwave extraction and hydro distillation. Int J Food Prop. 20(Suppl. 1):306–315.

[CIT0148] Morteza-Semnani K, Ahadi H, Hashemi Z. 2016. The genus *Hymenocrater*: a comprehensive review. Pharm Biol. 54(12):3156–3163. 27363483 10.1080/13880209.2016.1197285

[CIT0149] Morteza-Semnani K, Saeedi M, Akbarzadeh M. 2012. Chemical composition and antimicrobial activity of the essential oil of *Hymenocrater calycinus* (Boiss.) Benth. J Essent Oil-Bear Plants. 15(5):708–714.

[CIT0150] Mottaghipisheh J, Kiss T, Tóth B, Csupor D. 2020. The *Prangos* genus: a comprehensive review on traditional use, phytochemistry, and pharmacological activities. Phytochem Rev. 19(6):1449–1470.

[CIT0151] Mozafarian V. 1996. Dictionary of Iranian plant names. Tehran, Iran: Farhang Moasser Publisher.

[CIT0152] Mumivand H, Aghemiri A, Aghemiri A, Morshedloo MR, Nikoumanesh K. 2019. *Ferulago angulata* and *Tetrataenium lasiopetalum*: essential oils composition and antibacterial activity of the oils and extracts. Biocatal Agric Biotechnol. 22:101407–101430.

[CIT0153] Naghibi F, Mosaddegh M, Mohammadi Motamed M, Ghorbani A. 2005. Labiatae family in folk medicine in Iran: from ethnobotany to pharmacology. Iran J Pharm Res. 4:63–79.

[CIT0154] Najafi S, Nejad BS, Deokule S, Estakhr J. 2010. Phytochemical screening of *Bidaria khandalense* (Sant.) *Loranthus capitellatus* Wall., *Viscum articulatum* Burm. f. and *Vitex negundo* Linn. Res J Pharm Biol Chem Sci. 1:388–393.

[CIT0155] Nasab FK, Khosravi AR. 2014. Ethnobotanical study of medicinal plants of Sirjan in Kerman Province. J Ethnopharmacol. 154(1):190–197. 24746480 10.1016/j.jep.2014.04.003

[CIT0156] Nasseri M, Golmohammadzadeh S, Arouiee H, Jaafari MR, Neamati H. 2016. Antifungal activity of *Zataria multiflora* essential oil-loaded solid lipid nanoparticles *in vitro* condition. Iran J Basic Med Sci. 19:1231–1237. 27917280 PMC5126225

[CIT0157] Newman DJ, Cragg GM. 2012. Natural products as sources of new drugs over the 30 years from 1981 to 2010. J Nat Prod. 75(3):311–335. 22316239 10.1021/np200906s PMC3721181

[CIT0221] Nickavar B, Adeli A, Nickavar A. 2014. Analyses of the essential oil from *Bunium persicum* fruit and its antioxidant constituents. J Oleo Sci. 63(7):741–746. 24919477 10.5650/jos.ess13168

[CIT0158] Noudeh GD, Sharififar F, Noodeh AD, Moshafi MH, Afzadi MA, Behravan E, Aref M, Sakhtianchi R. 2010. Antitumor and antibacterial activity of four fractions from *Heracleum persicum* Desf. and *Cinnamomum zeylanicum* Blume. J Med Plant Res. 4:2176–2180.

[CIT0159] Omidpanah N, Valifard M, Esmaeili M, Yousefi R, Moghadam A. 2015. Antioxidant and antibacterial properties of the essential oils of two Iranian medicinal plants: *Zhumeria majdae* and *Salvia mirzayanii*. J Adv Med Sci Appl Technol. 1(1):51–60.

[CIT0160] Oroojalian F, Kasra-Kermanshahi R, Azizi M, Bassami MR. 2010. Phytochemical composition of the essential oils from three Apiaceae species and their antibacterial effects on food-borne pathogens. Food Chem. 120(3):765–770.

[CIT0161] Owfi R, Safaian N. 2017. Overview of important medicinal plants at Fars province. Iran Med Aromat Plants. 6:4–12.

[CIT0162] Pan SY, Litscher G, Gao SH, Zhou SF, Yu ZL, Chen HQ, Zhang SF, Tang MK, Sun JN, Ko KM. 2014. Historical perspective of traditional indigenous medical practices: the current renaissance and conservation of herbal resources. Evid Based Complement Alternat Med. 2014:525340–525361. 24872833 10.1155/2014/525340 PMC4020364

[CIT0163] Pauli A, Kubeczka KH. 2010. Antimicrobial properties of volatile phenylpropanes. Nat Prod Commun. 5(9):1387–1394. 20922996

[CIT0164] Pezhmanmehr M, Hasani ME, Jahansouz F, Najafi AA, Sefidkon F, Mardi M, Pirseyedi M. 2009. Assessment of genetic diversity in some Iranian populations of *Bunium persicum* using RAPD and AFLP markers. Iran J Biotechnol. 7:93–100.

[CIT0165] Poljsak B, Šuput D, Milisav I. 2013. Achieving the balance between ROS and antioxidants: when to use the synthetic antioxidants. Oxid Med Cell Longev. 2013:956792–956803. 23738047 10.1155/2013/956792 PMC3657405

[CIT0166] Prestinaci F, Pezzotti P, Pantosti A. 2015. Antimicrobial resistance: a global multifaceted phenomenon. Pathog Glob Health. 109(7):309–318. 26343252 10.1179/2047773215Y.0000000030 PMC4768623

[CIT0167] Raeisi M, Tajik H, Rohani SMR, Tepe B, Kiani H, Khoshbakht R, Aski HS, Tadrisi H. 2016. Inhibitory effect of *Zataria multiflora* Boiss. essential oil, alone and in combination with monolaurin, on listeria monocytogenes. Vet Res Forum. 7:7–11. 27226881 PMC4867031

[CIT0168] Rahimi V, Hekmatimoghaddam S, Jebali A, Khalili Sadrabad E, Akrami Mohajeri F. 2019. Chemical composition and antifungal activity of essential oil of *Zataria Multiflora*. J Nut Food Secur. 4:1–6.

[CIT0169] Rahimmalek M, Bahreininejad B, Khorrami M, Tabatabaei BE. 2009. Genetic variability and geographic differentiation in *Thymus daenensis* subsp. daenensis, an endangered medicinal plant, as revealed by inter simple sequence repeat (ISSR) markers. Biochem Genet. 47(11–12):831–842. 19657729 10.1007/s10528-009-9281-z

[CIT0170] Rahnama M, Najimi M, Ali S. 2012. Antibacterial effects of *Myristica fragrans*, *Zataria multiflora* Boiss., *Syzygium aromaticum*, and *Zingiber officinale* Rosci essential oils, alone and in combination with nisin on *Listeria monocytogenes*. Comp Clin Pathol. 21(6):1313–1316.

[CIT0171] Rakotoarivelo NH, Rakotoarivony F, Ramarosandratana AV, Jeannoda VH, Kuhlman AR, Randrianasolo A, Bussmann RW. 2011. Medicinal plants used to treat the most frequent diseases encountered in Ambalabe rural community, Eastern Madagascar. J Ethnobiol Ethnomed. 11:68–84. 10.1186/s13002-015-0050-2 PMC4570514 26369781

[CIT0223] Rao J, Chen B, McClements DJ. 2019. Improving the efficacy of essential oils as antimicrobials in foods: Mechanisms of action. Annu Rev Food Sci Technol. 10:365–387. 30653350 10.1146/annurev-food-032818-121727

[CIT0172] Rashidipour M, Ezatpour B, Talei GR, Pournia Y. 2016. The carvacrol level and antibacterial properties of industrial and laboratory essential oils of the wild and cultivated *Satureja khuzestanica*. J Essent Oil-Bear Plants. 19(3):519–528.

[CIT0173] Rasooli A, Fatemi F, Akbarzadeh K, Dini S, Bahremand S. 2016. Synergistic protective activity of deuterium depleted water (DDW) and *Satureja rechingeri* essential oil on hepatic oxidative injuries induced by acetaminophen in rats. J Essent Oil-Bear Plants. 19(5):1086–1101.

[CIT0174] Razzaghi-Abyaneh M, Shams-Ghahfarokhi M, Rai M. 2013. Antifungal plants of Iran: an insight into ecology. Chemistry and molecular biology. Heidelberg, Berlin: Springer Publisher.

[CIT0175] Rechinger K, Wendelbo P. 1967. Zhumeria majdae. Nytt Mag Bot. 14:39–43.

[CIT0176] Rechinger K. 1982. Flora Iranica, Labiatae. Akad Druke-u Verlag. 150:479–480.

[CIT0177] Rezayan A, Ehsani A. 2015. Evaluation of the chemical compounds and antibacterial properties of the aerial parts of Persian *Heracleum persicum* essence. J Babol Univ Med Sci. 17:26–32.

[CIT0178] Roshanaei K, Dadkhah A, Fatemi F, Dini S. 2017. *Heracleum persicum* essential oil administration in CCL4 treated rat sustains antioxidant/oxidative stress statue. Adv Biores. 8:93–101.

[CIT0179] Rustaie A, Keshvari R, Samadi N, Khalighi-Sigaroodi F, Ardekani MR, Khanavi M. 2016. Essential oil composition and antimicrobial activity of the oil and extracts of *Bunium persicum* (Boiss.) B. Fedtsch.: wild and cultivated fruits. Pharm Sci. 22(4):296–301.

[CIT0180] Sadat-Hosseini M, Farajpour M, Boroomand N, Solaimani-Sardou F. 2017. Ethnopharmacological studies of indigenous medicinal plants in the south of Kerman, Iran. J Ethnopharmacol. 199:194–204. 28167292 10.1016/j.jep.2017.02.006

[CIT0181] Saei-Dehkordi S, Fallah AA, Heidari-Nasirabadi M, Moradi M. 2012. Chemical composition, antioxidative capacity and interactive antimicrobial potency of *Satureja khuzestanica* Jamzad essential oil and antimicrobial agents against selected food-related microorganisms. J Food Sci Technol. 47(8):1579–1585.

[CIT0182] Saeidi M, Asili J, Emami SA, Moshtaghi N, Malekzadeh-Shafaroudi S. 2019. Comparative volatile composition, antioxidant and cytotoxic evaluation of the essential oil of *Zhumeria majdae* from south of Iran. Iran J Basic Med Sci. 1:80–85. 10.22038/ijbms.2018.20829.5418 PMC6437462 30944712

[CIT0183] Safa O, Soltanipoor MA, Rastegar S, Kazemi M, Dehkordi KN, Ghannadi A. 2013. An ethnobotanical survey on Hormozgan Province. Iran J Phytomed. 3:64–81. PMC4075690 25050260

[CIT0184] Sahari Moghadam A, Mehrafarin A, Naghdi Badi H. 2017. Chemical composition and antioxidant activity *Achillea millefolium* L. essential oils. Essent Oil-Bear Plants. 20(1):293–297.

[CIT0185] Saharkhiz M, Motamedi M, Zomorodian K, Pakshir K, Miri R, Hemyari K. 2012. Chemical composition, antifungal and antibiofilm activities of the essential oil of *Mentha piperita* L. ISRN Pharm. 2012:718645. 23304561 10.5402/2012/718645 PMC3532871

[CIT0186] Saharkhiz MJ, Zomorodian K, Taban A, Pakshir K, Heshmati K, Rahimi MJ. 2016. Chemical composition and antimicrobial activities of three *Satureja* species against food-borne pathogens. J Essent Oil-Bear Plants. 19(8):1984–1992.

[CIT0187] Saidi M. 2014. Antioxidant activities and chemical composition of essential oils from *Satureja khuzestanica*, *Oliveria decumbens* and *Thymus daenensis*. J Essent Oil-Bear Plants. 17(3):513–521.

[CIT0188] Sajed H, Sahebkar A, Iranshahi M. 2013. *Zataria multiflora* Boiss. (Shirazi thyme)—an ancient condiment with modern pharmaceutical uses. J Ethnopharmacol. 145(3):686–698. 23266333 10.1016/j.jep.2012.12.018

[CIT0189] Sayhoon M, Rajaie R, Hosseini SL, Nasiri SS, Sarabi M. 2013. Investigation of antibacterial activity of essential oil of gamma irradiated caraway seeds (*Carum carvi* L.). J Nucl Sci Technol. 1:60–64.

[CIT0225] Sharafati Chaleshtori F, Saholi M, Sharafati Chaleshtori R. 2018. Chemical composition, antioxidant and antibacterial activity of *Bunium persicum*, *Eucalyptus globulus*, and Rose Water on multidrug-resistant *Listeria* species. J Evid Based Integr Med. 23:1–7. 10.1177/2515690X17751314 PMC5871051 29405759

[CIT0190] Seidi Damyeh MS, Niakousari M, Saharkhiz MJ. 2016. Ultrasound pretreatment impact on *Prangos ferulacea* Lindl. and *Satureja macrosiphonia* Bornm. essential oil extraction and comparing their physicochemical and biological properties. Ind Crops Prod. 87:105–115.

[CIT0191] Seidi Damyeh MS, Niakousari M. 2016. Impact of ohmic-assisted hydrodistillation on kinetics data, physicochemical and biological properties of *Prangos ferulacea* Lindle. essential oil: comparison with conventional hydrodistillation. Innov Food Sci Emerg Technol. 33:387–396.

[CIT0192] Senatore F, Urrunaga Soria E, Urrunaga Soria R, Della Porta G, De Feo V. 1998. Essential oils from two Peruvian *Satureja* species. Flavour Fragr J. 13(1):1–4.

[CIT0193] Shafaghat A, Shafaghatlonba M. 2011. Comparison of biological activity and chemical constituents of the essential oils from leaves of *Thymus caucasicus*, *T. kotschyanus* and *T. vulgaris*. J Essent Oil-Bear. 14(6):786–791.

[CIT0194] Shahabi N, Tajik H, Moradi M, Forough M, Ezati P. 2017. Physical, antimicrobial and antibiofilm properties of *Zataria multiflora* Boiss. essential oil nanoemulsion. Int J Food Sci Technol. 52(7):1645–1652.

[CIT0195] Shahbazi Y, Shavisi N, Karami N, Kakaei S. 2015. Chemical composition and *in vitro* antibacterial activity of *Ferulago angulata* (Schlecht.) Boiss essential oil. Pharm Sci. 21(1):6–11.

[CIT0196] Shahbazi Y, Shavisi N, Modarresi M, Karami N. 2016. Chemical composition, antibacterial and antioxidant activities of essential oils from the aerial parts of *Ferulago angulata* (Schlecht.) Boiss and *Ferulago bernardii* Tomk. & M. Pimen from different parts of Iran. J Essent Oil-Bear Plants. 19(7):1627–1638.

[CIT0197] Shahbazi Y. 2015. Chemical composition and *in vitro* antibacterial effect of *Ziziphora clinopodioides* essential oil. Pharm Sci. 21(2):51–56.

[CIT0198] Shahbazi Y. 2017. Chemical compositions, antioxidant and antimicrobial properties of *Ziziphora clinopodioides* Lam. essential oils collected from different parts of Iran. J Food Sci Technol. 54(11):3491–3503. 29051644 10.1007/s13197-017-2806-2 PMC5629158

[CIT0199] Shakib P, Taherikalani M, Ramazanzadeh R. 2018. Chemical composition, genotoxicity and antimicrobial activities of *Dracocephalum kotschyi* Boiss. against OXA-48 producing *Klebsiella pneumoniae* isolated from major hospitals of Kurdistan Province. Microbiol Res J Int. 24(3):1–8.

[CIT0224] Sharififar F, Derakhshanfar A, Dehghan-Nudeh G, Abbasi N, Abbasi R, Gharaei RR, Koohpayeh A. 2011. In vivo antioxidant activity of Zataria multiflora Boiss. essential oil. Pak J Pharm Sci. 24:221–225. 21454174

[CIT0200] Shariatifar N, Mostaghim T, Afshar A, Mohammadpourfard I, Sayadi M, Rezaei M. 2017. Antibacterial properties of essential oil of *Heracleum persicum* (Golpar) and food-borne pathogens. Int J Enteric Pathog. 5(2):41–44.

[CIT0201] Sharma P, Jha AB, Dubey RS, Pessarakli M. 2012. Reactive oxygen species, oxidative damage, and antioxidative defense mechanism in plants under stressful conditions. J Bot. 2012:217037–217063.

[CIT0202] Sheibani M, Nayernouri T, Dehpour A. 2018. Herbal medicines and other traditional remedies in Iran—a tragedy unfolds. Arch Iran Med. 21(7):312–314. 30041530

[CIT0203] Sheikholeslami S, Mousavi SE, Ashtiani HR, Doust SR, Rezayat SM. 2016. Antibacterial activity of silver nanoparticles and their combination with *Zataria multiflora* essential oil and methanol extract. Jundishapur J Microbiol. 9(10):e36070. 27942360 10.5812/jjm.36070 PMC5136444

[CIT0204] Srinivasan K. 2018. Cumin (*Cuminum cyminum*) and black cumin (*Nigella sativa*) seeds: traditional uses, chemical constituents, and nutraceutical effects. J Food Saf. 2(1):1–16.

[CIT0205] Stevanovic ZD, Sieniawska E, Glowniak K, Obradovic N, Pajic-Lijakovic I. 2020. Natural macromolecules as carriers for essential oils: from extraction to biomedical application. Front Bioeng Biotechnol. 8:563–587. 32671026 10.3389/fbioe.2020.00563 PMC7330110

[CIT0206] Swamy MK, Akhtar MS, Sinniah UR. 2016. Antimicrobial properties of plant essential oils against human pathogens and their mode of action: an updated review. Evid Based Complement Alternat Med. 2016:3012462–3012483. 28090211 10.1155/2016/3012462 PMC5206475

[CIT0207] Taherpour AA, Khaef S, Yari A, Nikeafshar S, Fathi M, Ghambari S. 2017. Chemical composition analysis of the essential oil of *Mentha piperita* L. from Kermanshah, Iran by hydrodistillation and HS/SPME methods. J Anal Sci Technol. 8(1):1–6.

[CIT0208] Taherpour AA, Maroofi H, Changizi M, Shoushtari RV, Larijani K, Kazempour A. 2011. Chemical compositions of the essential oil and calculation the biophysicochemical coefficients of the components of *Hymenocrater longiflorus* Benth. of Iran. Nat Sci. 3(2):104–108.

[CIT0209] Tavakoli HR, Mashak Z, Moradi B, Sodagari HR. 2015. Antimicrobial activities of the combined use of *Cuminum cyminum* L. essential oil, nisin and storage temperature against *Salmonella typhimurium* and *Staphylococcus aureus in vitro*. Jundishapur J Microbiol. 8(4):e24838–e24845. 26034554 10.5812/jjm.8(4)2015.24838 PMC4449852

[CIT0210] Veeresham C. 2012. Natural products derived from plants as a source of drugs. J Adv Pharm Technol Res. 3(4):200–201. 23378939 10.4103/2231-4040.104709 PMC3560124

[CIT0211] Yazdani M, Jookar Kashi F, Dashti Zadeh Z. 2019. Evaluation of antimicrobial and antioxidant activity of essential oil of *Mentha piperita* L. Iran J Med Microbiol. 13(3):210–219.

[CIT0212] Yousefi K, Hamedeyazdan S, Hodaei D, Lotfipour F, Baradaran B, Orangi M, Fathiazad F. 2017. An *in vitro* ethnopharmacological study on *Prangos ferulacea*: a wound healing agent. Bioimpacts. 7(2):75–82. 28752071 10.15171/bi.2017.10 PMC5524988

[CIT0213] Zinoviadou KG, Koutsoumanis KP, Biliaderis CG. 2009. Physico-chemical properties of whey protein isolate films containing oregano oil and their antimicrobial action against spoilage flora of fresh beef. Meat Sci. 82(3):338–345. 20416718 10.1016/j.meatsci.2009.02.004

[CIT0214] Zolfaghari MR, Jalali Yazdi A, Fatemi F. 2015. Effect of γ-irradiation on the antibacterial activities of *Cuminum cyminum* L. essential oils *in vitro* and in *in vivo* systems. J Essent Oil-Bear Plants. 18:582–591.

[CIT0215] Zomorodian K, Ghadiri P, Saharkhiz MJ, Moein MR, Mehriar P, Bahrani F, Golzar T, Pakshir K, Fani MM. 2015. Antimicrobial activity of seven essential oils from Iranian aromatic plants against common causes of oral infections. Jundishapur J Microbiol. 8(2):e17766. 25793100 10.5812/jjm.17766 PMC4353034

[CIT0216] Zomorodian K, Moein M, Pakshir K, Karami F, Sabahi Z. 2017. Chemical composition and antimicrobial activities of the essential oil from *Salvia mirzayanii* leaves. Evid Based Complement Alternat Med. 22(4):770–776. 10.1177/2156587217717414 PMC5871294 28689440

